# Antibiotic resistance and host immune system-induced metal bactericidal control are key factors for microbial persistence in the developing human preterm infant gut microbiome

**DOI:** 10.3389/fmicb.2022.958638

**Published:** 2022-11-21

**Authors:** Samantha L. Peters, Michael J. Morowitz, Robert L. Hettich

**Affiliations:** ^1^Biosciences Division, Oak Ridge National Laboratory, Oak Ridge, TN, United States; ^2^Graduate School of Genome Science and Technology, The University of Tennessee, Knoxville, TN, United States; ^3^Department of Surgery, University of Pittsburgh School of Medicine, Pittsburgh, PA, United States

**Keywords:** human microbiome, human infant faecal microbiota, Metaproteomics, host immune system, metal bactericidal control, antibiotic resistance

## Abstract

The human gut microbiome, which develops and stabilizes during the early stages of infant life, plays an essential role in host health through the production of metabolic resources and the stimulation and training of the immune system. To study colonization and community functional dynamics of the microbiota based on responses to host immune processes during the normal and dysbiotic establishment of the gut, metaproteomics was conducted on 91 fecal samples collected over the first 90 days of life from 17 hospitalized premature infants. Microbial responses to antibiotic administration and host-imposed metal bactericidal control correlated with community assembly and resiliency of microbes in the developing preterm gut. Specifically, proteins related to antibiotic resistance and metal homeostasis mechanisms were predominant in persisting members in the infant gut environment over the first several weeks of life. Overall, this metaproteomics study provides a unique approach to examine the temporal expansion and resilience of microbial colonization, as it allows simultaneous examination of both host and microbial metabolic activities. Understanding the interplay between host and microbes may elucidate the microbiome’s potential immunomodulatory roles relevant to necrotizing enterocolitis and other dysbiotic conditions in preterm infants.

## Introduction

Early-life microbial colonization of mucosal surfaces in the gut during infancy is critical for balanced priming and education of the host immune system. Recent studies are defining the period in early development, a so-called “window of opportunity,” where disruption of critical host-commensal interactions have lasting and sometimes irreversible impacts on the training of specific immune subset (Gensollen et al., 2016). Studies using rodent models have shown that a brief, postnatal germfree period caused permanent changes in levels of systemic regulatory T cells, natural killer cells, and cytokine production (Hansen et al., 2012) and that microbial exposure during early life has persistent effects on natural killer T cell function, which was shown to be crucial in the development of intestinal inflammatory disorders such as colitis (Olszak et al., 2012). Other intestinal inflammatory diseases, such as necrotizing enterocolitis, can result from exaggerated immune responses (Buttó et al., 2015; Mihi and Good, 2019). Necrotizing enterocolitis (NEC) is a fatal disease of neonatal preterm infants in 30–50% of cases (Madden, 2021). It is associated with intestinal inflammation driven by the microbiota, making it an ideal model for studying early-life host–microbe interactions in a dysbiotic gut (Hackam and Caplan, 2018; Gopalakrishna et al., 2019). Most interactions between microbiota and the host immune system occur on the mucosal surface of the large intestines. This surface is lined with intestinal epithelial cells (IECs), which create a physical barrier between the microbiota in the intestinal lumen and the surrounding tissue. IECs secrete antimicrobial peptides such as defensins, and also secrete cytokines and chemokines that modulate immune function to help prevent bacterial from breaching the gut wall (Mirzaei and Maurice, 2017). Bacterial toxins and pathogen-associated molecular patterns (PAMPs), including bacterial flagellin, unmethylated CpG oligodeoxynucleotides, lipopolysaccharide (LPS), lipoteichoic acid, are recognized by host toll-like receptors, which can activate pro-inflammatory responses. There is a delicate balance related to these host–microbe interactions in order to maintain homeostasis in the environment. However, the underpinnings of this balance are largely unknown, especially related to the temporal aspects of host–microbe interactions during the development of dysbiosis and inflammation. It is still unclear whether microbial community and functional shifts precede inflammation or are a consequence of it.

In addition to the still poorly understood interplay between the host immune system and microbiota, other external factors, such as antibiotic administration impact the developing gut environment. Preterm infants commonly receive antibiotics early and frequently in life due to their vulnerability to infection (Gibson et al., 2016; Gasparrini et al., 2019). Previous metagenomics studies have shown enrichment in antibiotic resistance genes in preterm infants that receive early-life antibiotics (Gasparrini et al., 2019; Chen et al., 2020). This perturbation of optimal microbiota colonization likely has long-term impacts on microbial establishment patterns and, ultimately, host health outcomes (Rodríguez et al., 2015; Healy et al., 2022). While host health outcomes based on gut microbial perturbations caused by infant antibiotic administration have been widely demonstrated (Fouhy et al., 2012), the exact mechanism by which it plays on the priming of the developing immune system is still unclear.

Several studies have shown that antibiotic resistance is linked to tolerance of high environmental concentrations of metals such as copper (Cheng et al., 2019; Pietsch et al., 2020). The phenomenon of co-resistance to antibiotics and excess metals has been observed in several organisms commonly found in the human gut (Hasman and Aarestrup, 2002; Hasman et al., 2006; Touati et al., 2010; Gómez-Sanz et al., 2013; Mourão et al., 2015), including one study that reported that multi-drug-resistant Enterobacterales carried metal resistance genes up to seven times more frequently compared to antibiotic-sensitive strains (Yang et al., 2018). Disrupting the availability of metals in the environment is an evolutionarily ancient strategy to limit bacterial growth, and vertebrates have developed immune-related mechanisms, in processes referred to as nutritional immunity, that restrict the availability of some metals to microbiota in the environment while at the same time flooding infection sites with antimicrobial levels of other metals (Becker and Skaar, 2014; Djoko et al., 2015). Concurrently, many organisms have developed mechanisms to maintain intracellular metal homeostasis, including resistance to excess environmental concentrations, as the balance of metals in the cell is necessary to prevent mismetallation and cell damage (Lemire et al., 2013; Waters, 2020). *In vivo* characterization of metal homeostasis in the gut environment is challenging due to the technical limitations of most traditional microbiome research methods. Nevertheless, this is an understudied area that needs to be considered for its unrecognized role in shaping the early life gut.

To this end, this study was designed to examine the gut microbiota response to human host-imposed immune control during establishment of the developing gut microbiome over the first few months of life, specifically utilizing a high-resolution LC–MS/MS approach that enables simultaneous investigation of host and microbial functions that impact this colonization process. This was based on a combined, longitudinal metagenomics and metaproteomics study of fecal microbiome samples collected from hospitalized premature infants, including a subset of infants who developed NEC during the study. The developing infant gut is an ideal model system because infant microbiomes are far less complex than adult microbiomes. The early-life period is critical in the maturation of both the immune system and microbial communities. Earlier investigations on a subset of the samples used in the present analysis predominantly focused on microbial metabolic functions and community composition (Xiong et al., 2017; Brown et al., 2018). These investigations led to a few key findings that expand understanding of the establishment dynamics in the early-life preterm gut. First, the initial investigation focused on four of the infants used in the present analysis. The initial analysis demonstrated that the premature infant microbiome is highly variable, with metaproteome measurements showing drastic differences in functional composition between infants and across time. Second, following these findings, an expanded analysis was conducted for paired metagenomic and metaproteomic measurements for samples collected from 15 infants, which revealed that genetically similar organisms colonizing different infants have very distinct proteomes with unique metabolic profiles, supporting the idea that microbiome function largely depends on microbial responses to the physiological conditions present at a given point in time in the infant gut and is not solely dependent upon which organisms are present. Third, microbiome diversity was lower in samples collected during or within 5 days of antibiotic treatment. Using replication rates and overall abundances of organisms within these infants demonstrated that some organisms in the preterm infant gut persisted and continued replicating in the presence of administered antibiotics, indicating some level of resistance to those antibiotics. Finally, in several instances, there were shifts in both taxonomy and microbial function preceding NEC diagnosis; however, no specific species or metaproteome type could explain all infants who developed this condition during the study. However, these works largely ignored the contribution of the host immune system, which plays a crucial role in shaping the early-life gut environment (Hansen et al., 2012; Melville and Moss, 2013; Kai-Larsen et al., 2014; Busi et al., 2021; Henderickx et al., 2021), although the exact mechanisms of this process are still poorly understood. A clear understanding of host–microbe interactions during the colonization process depends upon the intrinsic capabilities of metaproteomics measurements to capture host and microbiota functional information simultaneously and link measured entities back to source organisms. In the present analysis, we set out to elucidate the host immune system’s specific roles on microbial establishment dynamics and to study how microbes modulate their function during the colonization process in response to and as a stimulant of the developing host immune system.

## Results

### Metaproteomics measurements reveal simultaneous information about temporally-connected host immune processes and microbial functions in the fecal samples

**Figure 1:**
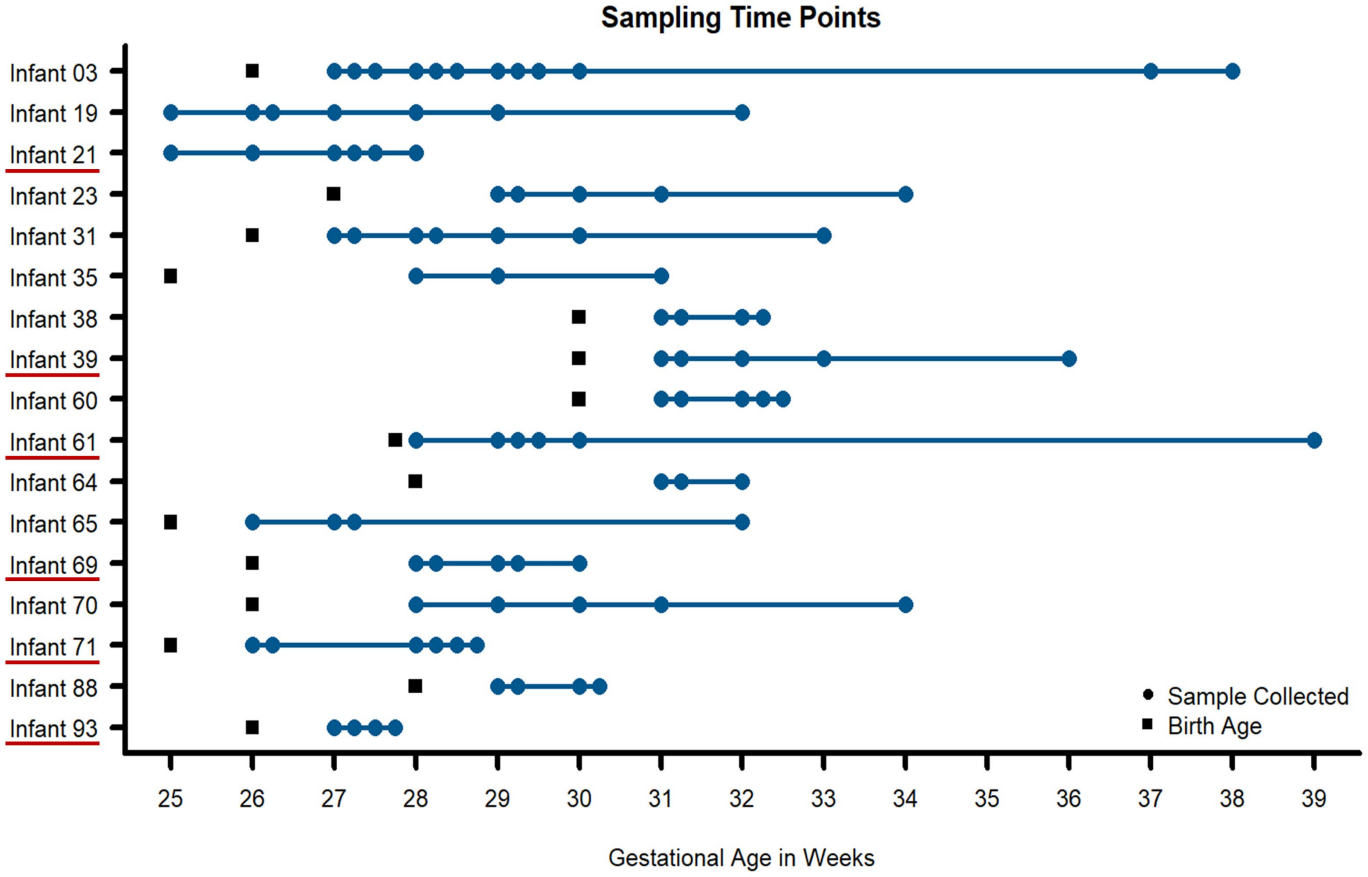
Fecal samples collected for metaproteomic measurements for all infants in the study. Using necrotizing enterocolitis (NEC) as a representative dysbiotic condition, 91 fecal samples (blue circles) were taken from 17 preterm infants over the first 90 days of life. Multiple time points were measured per infant to assess intraindividual variability over time. Six infants developed NEC during the early stages of life (underlined in red on the y-axis). Birth age (in gestational weeks) is noted for each infant with black squares.

**Figure 2:**
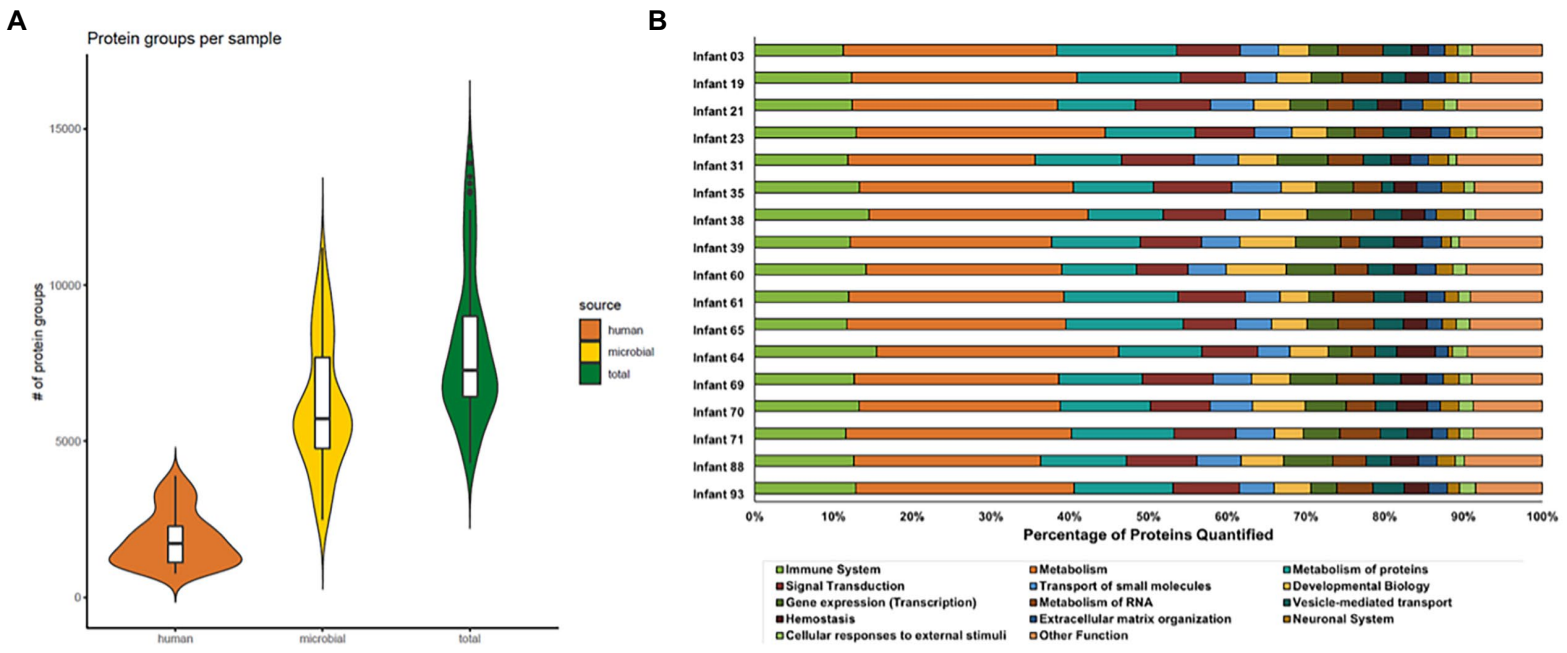
Distribution of protein groups identified in each sample for each protein source **(A)** and the percentage of human proteins quantified in each Reactome annotation category **(B)**. In panel A, protein group identifications were based on uniquely mapping peptides. Microbial and human proteins were clustered at 100 and 85% sequence similarity, respectively. Each violin plot illustrates the kernel probability density for all samples collected (the width represents the proportion of the data) and the horizontal bars on the box plot depict the median and interquartile range of the distribution. In panel B, the Reactome database was used to annotate human proteins based on proteins that uniquely map to one category. The bar plots show the proportion of proteins in each Reactome category quantified per infant. Among the quantified human proteins, proteins related to immune functions (lime green bars) represent around 10–15% of human protein identifications.

Samples were collected during the first 3 months of life from hospitalized premature infants, including six infants that went on to develop necrotizing enterocolitis (NEC) during the study. In total, 91 stool samples from 17 infants were measured and analyzed (Figure 1). The sample selection for this study was heavily constrained by clinical factors rather than optimum experimental design for this study and thus there were unavoidable differences between infants related to the number of samples collected and the times that samples were collected among infants in the study. Across the entire dataset, 595,324 peptide sequences were measured across all 91 samples. This equated to, on average, 2000 human and 5,000 microbial protein groups quantified per sample, respectively (Figure 2A, Supplemental Table 1). Although a newer distinct database searching strategy was utilized in the present study, the average number of peptides and protein groups identified per sample was similar to results obtained with the alternative approaches used in previous investigations (Xiong et al., 2017; Brown et al., 2018). In each sample, human and microbial protein group identifications had similar relative proportions, with microbial proteins composing ~70% of the identified protein groups. Although individuals with samples spanning an extensive time range had more unique protein identifications than infants with only a few samples collected over a very short period, at a higher level (pathways and protein classes), the functional categories were fairly uniform across samples from all infants. Among human protein groups, immune protein groups make up around 10–15% of relative abundance when looking at protein groups that uniquely mapped to one category (Figure 2B), with ~45% of quantified proteins are unique to each specific immune pathway (Supplemental Table 2).

### Functional β-diversity of host and microbial proteins reveals distinct partitioning which is quite variable and is highly infant-specific

To narrow the investigation to focus on biological drivers of microbiome function related to host immune mechanisms, we used non-metric multidimensional scaling of Jaccard distances to assess the functional β-diversity of samples based on the presence or absence of proteins quantified in each sample. In addition, Bray-Curtis distances were also assessed to account for protein abundance in addition to the presence or absence of proteins. The functional dissimilarities between samples were explored on four different levels: (Gensollen et al., 2016) all quantified human protein and microbial KEGG orthology (KO) terms, (Hansen et al., 2012) only microbial KO terms, (Olszak et al., 2012) all human proteins, and (Mihi and Good, 2019) only immune-related human proteins. In this study, NEC was used as a representative dysbiotic condition to compare normal and abnormal establishment of the gut environment. Due to the heterogeneity in colonization patterns among infants who did or did not go on to develop this condition, it is apparent that other health factors can also have critical impacts on both host and microbial protein expression during this phase of life. To explore the impact of these other factors, ordination analysis was paired with Linear mixed-effects models (LMMs) to see if any health metadata categories (Supplemental Table 3) were significant drivers of separation between samples. In this analysis, infant ID was specified as a random factor to account for any non-independence of data to avoid overestimation of effects due to pseudo-replication. This analysis showed that time (day of life or gestational age) was the primary factor influencing the functional β-diversity of samples for each infant (value of *p* <0.01). Other factors such as delivery mode, feeding type, and disease onset did not significantly influence the functional β-diversity in this sample cohort. To complement the analysis using LMMs, PERMANOVA and homogeneity of multivariate dispersion testing were conducted on the Jaccard and Bray-Curtis distances for each of the metadata categories investigated (Supplementary Figures S1–S20). The largest factor contributing to the variance between samples considering all four functional levels was the infant each sample was collected from, with more than 67% of the variance across all proteins measured in the dataset explained by this factor.

Due to the heterogeneity in infant health characteristics (delivery mode, gestational age, disease status, antibiotic administration, time of sample collection, etc.), many of the observed trends were largely infant-specific. Based on this level of global analysis, it was clear that the approach of evaluating all samples from the infants in the cohort together could lead to a confounded interpretation of the data, as host-microbial interactions are highly dependent on the environmental context at the time of sample collection. In addition, grouping samples without accounting for sampling time washed out some observations of highly dynamic, time-dependent processes.

The ability to interrogate these temporal dynamics was a key strength of this sample set, which led us to the conclusion that an alternative approach needed to be taken to analyze the data further. In light of this conclusion, we set out to investigate some of the unanswered questions that arose in previous analyses of this dataset related to antibiotic resistance and shifts in community abundance that were not associated with times of disease onset.

### Antibiotic resistance mechanisms help selected microbes overcome susceptibility to antibiotics and persist in the environment

Based on previous findings (Xiong et al., 2017; Brown et al., 2018), it was apparent that there were antibiotic resistance-related proteins in *E. faecalis* within some of the infants in this cohort and the replication rates of selected microbes during periods of antibiotic administration indicated antibiotic resistance might be highly prevalent in organisms dominating the early-life gut environment. However, protein expression of the specific antibiotic resistance mechanisms used by any microbes in this dataset was not interrogated. Here, we set out to determine the extent of antibiotic resistance in the gut microbiome for all infants in this cohort and to characterize the diversity of antibiotic resistance mechanisms being utilized by the microbiota. All quantified microbial proteins were searched against the comprehensive antibiotic resistance database (CARD). In total, 241 protein sequences were identified across all 17 infants that mapped to 93 antibiotic resistance ontology (ARO) terms (Supplemental Table 4). These proteins represented six different resistance mechanisms. This protein overlap illustrates the functional redundancy of gut microbiota—with several organisms simultaneously expressing the same antibiotic resistance protein. It also supports the previous inference that the infant’s gut is a reservoir for antibiotic resistance genes (Gosalbes et al., 2016; Nogacka et al., 2017; Healy et al., 2022).

**Figure 3:**
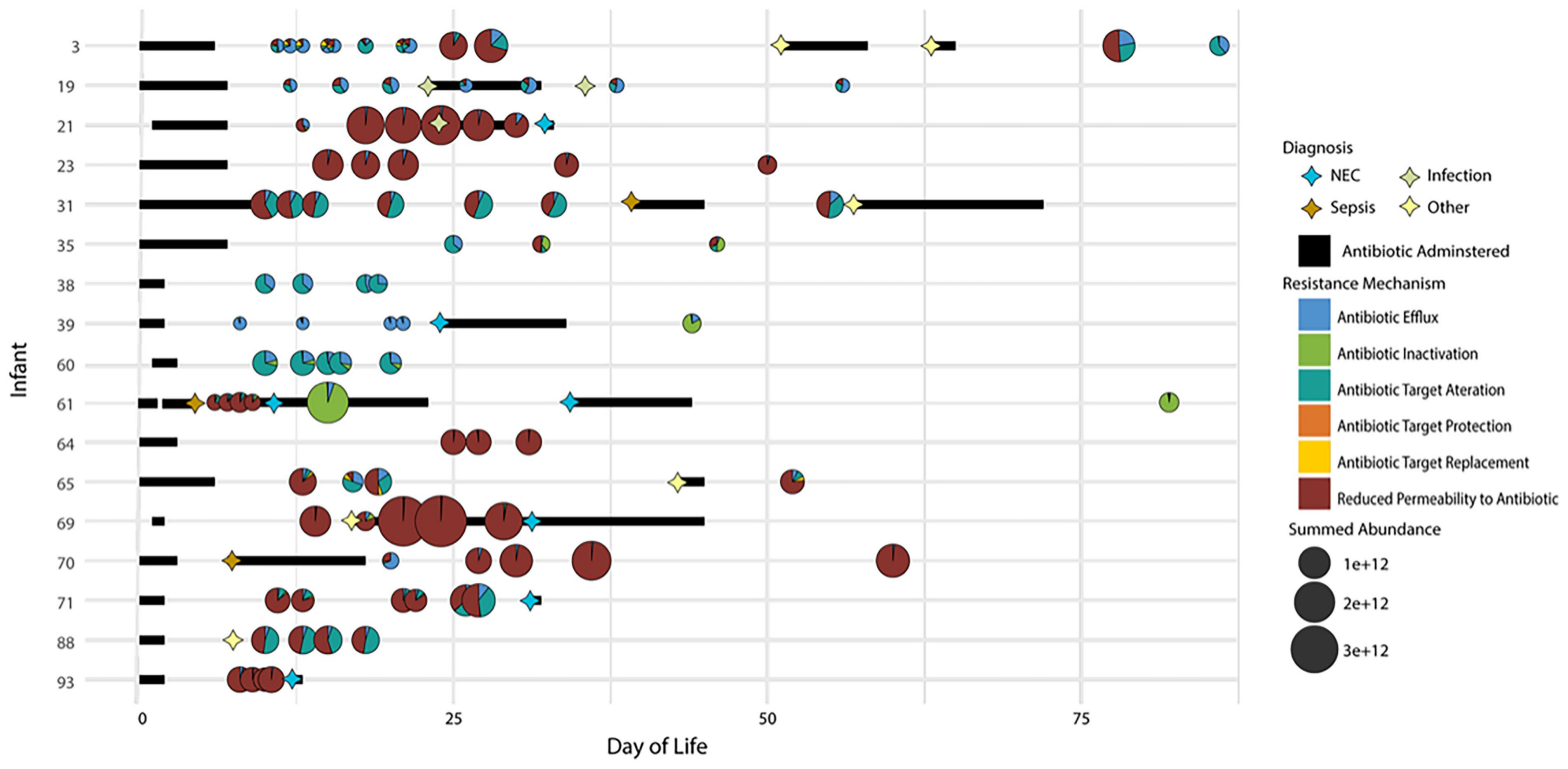
Distribution of antibiotic resistance orthologs (AROs) found in each sample for all 17 infants in the study. Samples are plotted based on time of collection (DOL) on the x-axis and by the infant on the y-axis. The size of the bubble indicated the summed ARO abundance in the sample. The pie charts indicate the contribution of each CARD database antibiotic resistance mechanism identified in the sample to the overall summed ARO abundance. Also plotted are markers indicating dates of disease diagnosis and bars to indicate periods of time when antibiotics were administered.

On average, 17 AROs were found in each timepoint across infants. In general, while the overall abundances of antibiotic resistance proteins changed over time, the relative contribution of individual resistance mechanisms utilized by microbiota remained unchanged across time in a specific individual unless perturbation of the system by administration of antibiotics. In addition, the overall ARO composition was highly variable among infants. Figure 3 depicts a summary of this information across all infants. Although this figure is somewhat complicated, it is an attempt to consolidate and compare the temporal aspects of disease diagnosis (including recurrence) and resistance mechanisms across all infants in one visual display. Among the entire infant cohort (Figure 3) only four infants (3, 35, 65, and 71) had evident variations in the resistance mechanisms over time without antibiotic administration. In these infants, over time there was a change in the types of resistance mechanisms being expressed that did not correspond with periods of administered antibiotics. In addition, in many samples, there was a change in the expression of AROs (based on summed abundance) that was not proportional to the change in the overall proteome abundance of the organisms expressing the ARO proteins at those times. This suggests that the observed increase or decrease in ARO expression is not caused by a corresponding increase or decrease in the relative abundance of ARO-producing organisms in the community. Overall, the antibiotic resistance patterns are quite variable over time, thus making it difficult to generalize conclusions across infants.

**Figure 4:**
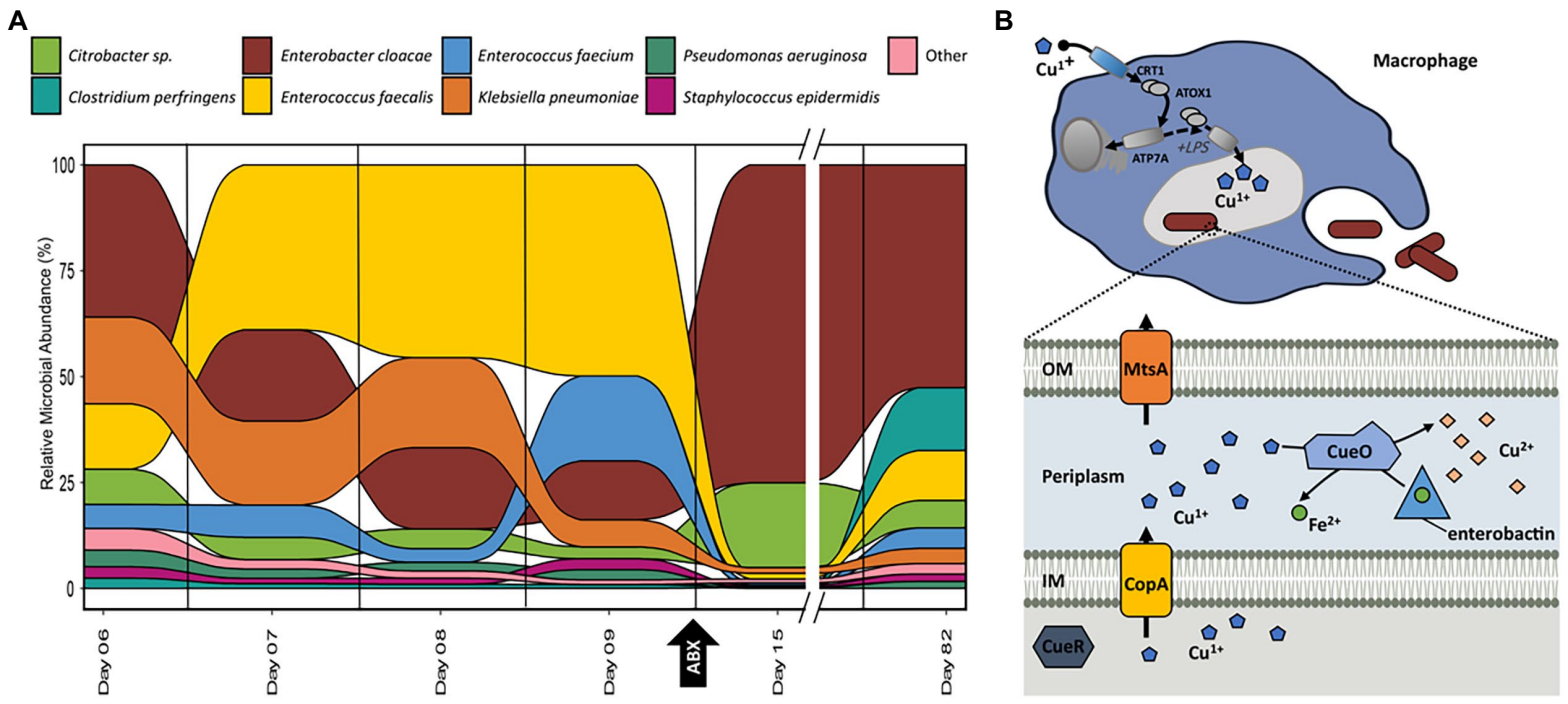
**(A)** Relative abundance of organisms at each time-point for samples collected for infant 61 based on the summed protein expression of each organism. Taxonomic assignment of proteins determined previously in Brown et al. (2018). **(B)** Graphical representation of copper/iron-related activities between the host immune system and *E. cloacae* in infant 61 based on the pathways and proteins detected in samples from this infant. Additional proteins related to these processes are listed in supplemental tables.

**Figure 5:**
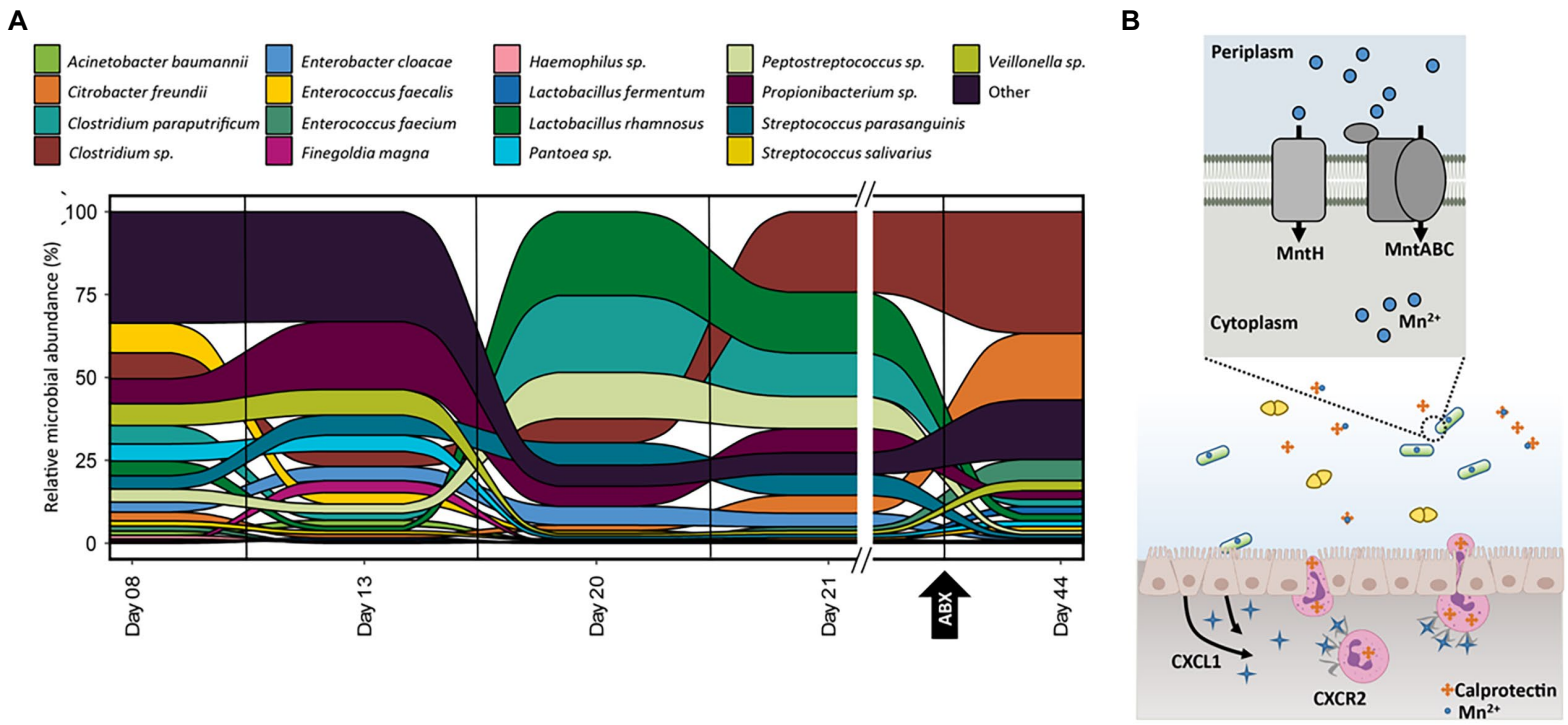
**(A)** Relative abundance of organisms at each time-point for samples collected for infant 39 based on the summed protein expression of each organism. Taxonomic assignment of proteins determined previously in Brown et al. (2018). **(B)** Graphical representation of manganese-related activities between the host immune system and *L. rhamnosus* in infant 39 based on the pathways and proteins detected in samples from this infant. Additional proteins related to these processes, as well as microbial manganese/zinc non-specific transporters, are listed in supplemental tables.

Upon examining the relative summed protein abundance of each organism in the community across the longitudinal series for each infant, some changes in community composition were observed, as would be expected based on antibiotic treatment of the infants over the first several weeks of life. In samples collected after the antibiotics were administered, there was a dramatic shift in the relative community composition based on this perturbation. The expression of some AROs with known resistance to the specific antibiotics administered at disease onset corresponded to the persistence of the microbiota expressing those genes at the time of antibiotic treatment. Several infants were treated with claforan upon diagnosis of sepsis or NEC. Among the infants with samples collected before and after antibiotic treatment, the expression of antibiotic inactivation related proteins by a microbe was indicative of that microbe’s persistence in the gut during periods of antibiotic administration. For example, in infants 61 and 39 (Figures 4A, 5A), claforan was one of several antibiotics administered at the time of NEC and/or sepsis diagnosis. Claforan should have been effective against the species present at the time of antibiotic treatment in these infants. However, there were several antibiotic resistance related genes that were being expressed by various organisms in this infant. Most of these genes were general RND-type antibiotic efflux pumps that have been associated with resistance to multiple antibiotic classes, including cephalosporins (the drug class of claforan). In both of these infants, only *Citrobacter* sp. were expressing CMY-type beta lactamases, which confer resistance to oxyimino-cephalosporins, including claforan. While the susceptibility to antibiotics and associated resistance mechanisms expressed by microbes played an essential role in establishment dynamics, several dramatic changes in organismal abundance were not explained by the expression, or lack thereof, of antibiotic resistance-related proteins in individual microbes. This indicates there must be other drivers of community establishment dynamics, and we hypothesized that microbial responses to host immune mechanisms might explain some shifts in relative organismal abundance across time in each infant.

Looking at samples across time within each individual yielded unique patterns of community dynamics in response to antibiotic administration and host processes when patient medical information was considered. Leveraging patient metadata regarding disease onset and the timing and types of antibiotics administered to each infant, functional shifts of individual microbiota could be differentiated between microbial responses to known and external perturbations on the gut environment caused by antibiotic administration and unknown and internal perturbations caused by interactions with host processes and other community members. While this type of analysis is highly individual-specific, it provides insights into community establishment dynamics that would otherwise be missed if samples from infants were generalized to broad analysis classes without accounting for the heterogeneity of all longitudinal and environmental factors which have a non-negligent impact on the colonization of the gut environment. To highlight this type of health-status-based longitudinal analysis, selected infants will be highlighted and discussed that have a wide range of sampling time points leading up to disease diagnosis and subsequent antibiotic treatment and samples collected during and after antibiotic administration. The observed protein expression profiles are not unique to these selected example infants but are found in multiple infants across the cohort.

### Metal homeostasis mechanisms help microbes overcome host-imposed copper toxicity and iron restriction

Out of the 17 infants in the cohort, infant 61 was the only infant diagnosed with sepsis and recurrent NEC (Figure 3). Several samples were collected from subsequent days prior to the initial NEC diagnosis as well as samples collected during and after antibiotic administration. This detailed sampling allowed us to examine the observed changes in microbial functional composition preceding antibiotic administration. In infant 61, after antibiotic treatment is initiated on day nine, there is a dramatic shift in the relative community functional composition based on this perturbation with both a reduction of some community members and a subsequent rebound in the relative abundance of those members observed after antibiotic treatment is completed (Figure 4A). However, not all changes in organismal abundance can be explained by susceptibility to antibiotics and associated resistance mechanisms being expressed. Among the three dominant organisms in this infant (*Enterococcus faecalis, Klebsiella pnuemoniae*, and *Enterobacter cloacae*), all three are typically susceptible to the types of antibiotics delivered during the course of the study. *E. faecalis* was the dominant organism, based on summed protein abundance, leading up to NEC diagnosis on day nine and decreased in relative abundance following subsequent initiation of antibiotic treatment. After 6 days of continuous antibiotics, it was drastically reduced in relative abundance, corresponding to its susceptibility to the antibiotics administered and the lack of resistance mechanisms. *E. cloacae* increased in relative abundance after the administration of antibiotics on day nine and persisted as the dominant organism in the community until the measurement on day 82. *K. pnuemoniae* was the dominant organism with protein expression relating to antibiotic resistance mechanisms (Supplemental Table 4). However, this organism’s relative abundance was already decreasing by day nine, before the start of antibiotic treatment, which indicates there are additional drivers of community establishment. We hypothesized that host immune responses might be contributing to the community shift.

Among host proteins related to immune response, there was increased host expression of proteins related to copper trafficking for antimicrobial activity starting at day eight. Expression of host proteins related to copper trafficking, including PDZD11 and binding partner PLEKHA5, was observed in five out of the six timepoints collected for infant 61. In addition, there was also evidence of host trafficking of iron observed in this infant, with all timepoints in this infant displaying the highest average abundance of the siderophore binding protein, lipocalin-2 (LCN2), observed across the entire infant cohort. LCN2 is known for its ability to bind bacterial siderophores, microbial proteins that assist in iron harvesting, such as enterobactin and salmochelin, to limit the bacterial iron acquisition and subsequently hamper the growth of microbes (Gerner et al., 2020).

Based on the indications that some infants were mounting an active immune response utilizing copper and iron trafficking-related antimicrobial activities, we focused on related metal trafficking activities in the microbiota. In each infant with increased host expression of proteins related to copper toxicity as an immune response, including infant 61, some microbes expressed proteins associated with copper toxicity resistance or tolerance, including CueR, CueO, and CopA. These proteins are specifically involved in the efflux of Cu (I) from the cell and the transformation of Cu (I) into Cu (II). In addition, there was expressional evidence of resistance-nodulation-division (RND) family transporters which many confer both metal and antibiotic resistance across the infant cohort. For example, proteins that comprise the cusABCF system were expressed by Gram negative bacteria in 11 of the 17 infants in the cohort. These resistance mechanisms may explains how some organisms within these infants might be persisting despite pressure from both host defences and antibiotic administration.

Comparing the functional activities of the individual microbes in this infant, we found that *E. cloacae* expressed many proteins related to copper efflux. While copper efflux-related proteins were expressed by some other bacteria in this infant during the sample collection period, fewer proteins related to this function were observed and they were in reduced abundance compared to *E. cloacae.* This suggests copper efflux and resistance were not as prominent in these organisms, even if there was an active response to excess copper. In addition, some of the proteins involved in the copper response identified in the two other dominant organisms do not have definitive physiological roles, unlike the proteins observed in *E. cloacae*, where there is a definitive path of efflux and detoxification of copper. Among the other dominant organisms in infant 61, *E. faecalis* was also found to express copper homeostasis-related proteins (CopA, CopB, and CutC), but at lower levels than *E. cloacae*. In addition to proteins involved in mitigating intracellular copper toxicity, only *E. cloacae* showed expression of multiple proteins related to the biosynthesis of the siderophore enterobactin among the organisms in this infant. As only *E. cloacae* was the only organism with observed expressional evidence of producing and importing enterobactin in this infant due to the elevated demand for iron-based on high levels of Cu (I) ions produced through host-defense mechanisms, the other species were compromised by their inability to offset Cu (I) levels intracellularly with iron and the lack of different mechanisms to export Cu (I).

### Host sequestration of manganese and zinc does not impact microbes with enhanced import capabilities

Infant 39 had multiple time points sampled across the first several weeks of life, with samples collected before and after NEC diagnosis and subsequent antibiotic administration. There was a shift in community structure at the time of NEC diagnosis on day 24 and the subsequent treatment, which we presume was predominantly caused by the administration of antibiotics (Figure 5A). For example, *C. freundii* expressed the majority of the antibiotic resistance-related proteins in this infant and dramatically increased in abundance after administration of these antibiotics. This supports the idea that organisms with antibiotic resistance mechanisms may persist and expand while the organisms lacking these mechanisms become dramatically reduced in abundance after antibiotic treatment. While the expression of antibiotic resistance genes by organisms during periods of antibiotic administration plays a role in shaping overall community composition, there are also changes in organismal abundance that are not correlated to microbial response to antibiotic treatment.

There was also an unexplained shift in community functional composition between samples on DOL 13 and 20. During this time period, there was a corresponding host expression of host immune proteins related to manganese and zinc trafficking. Two host proteins, S100A8 and S100A9, can be involved in metal sequestration by antimicrobial peptides and were found in high abundance across all 91 samples in the dataset. These two proteins form the heterodimer calprotectin, which is often used as a fecal biomarker of inflammation and sequesters both zinc (Zn) and manganese (Mn) when it is released by neutrophils at infection sites (Ho et al., 2019). In infant 39, calprotectin showed more than a three-fold increase during this period before returning to early-life levels at DOL 44. The most dramatically shifting organism between days 13 and 20 was *Lactobacillus rhamnosus*. We hypothesized that the dramatic increase and later decrease of calprotectin might be influencing the relative abundance of *L. rhamnosus* over the first several weeks of life. As calprotectin inhibits bacterial growth through chelation of manganese and zinc, we looked to associated mechanisms, such as membrane transporters, in *L. rhamnosus* related to manganese and zinc import that helped this organism import these metals into the cell to overcome the host-imposed metal limitation. This organism expressed four key proteins related to manganese trafficking; mntA, mntB, mntC, and mntH. The MntABC protein complex has been demonstrated to import manganese during low Mn supply, and mntH specifically competes with calprotectin for luminal manganese (Kehl-Fie et al., 2013). In addition to manganese-specific transport proteins, several proteins were either specific to zinc transport or related to the non-specific transport of zinc and manganese, which indicates the microbes in this gut environment were actively coping with zinc and manganese limitations in the surrounding environment*. L. rhamnosus* expressed non-specific zinc/manganese transport system substrate-binding proteins and a manganese-dependent inorganic pyrophosphatase (ppaC), which indicates the microbe was actively importing manganese and utilizing it within the cell. Notably, *L. rhamnosus* was also expressing a putative drug exporter of the RND superfamily, which may also contribute to the persistence of this organism during periods of antibiotic administration.

## Discussion

Across the entire dataset, there were 1,000s of microbial and human peptides measured per sample, which equated to an average of 3,000 human proteins and 9,000 microbial proteins identified per infant. This proteomic coverage achieved allowed in-depth elucidation of both host and microbiota function in the early-life preterm gut. Among the host proteins related to immune function, which represented 10–15% of human proteins quantified in the dataset, 40–50% of these quantified proteins were unique to specific immune pathways, which led to the confident interpretation of the host functional data related to the research questions addressed in this study. The functional β-diversity analysis showed that this intra-and inter-individual functional variability is not restricted to the microbiota. Host protein expression related to immune function was variable and dependent on the environmental context each infant faced during this early-life period. In spite of the inter-individual variability, the longitudinal measurements collected in this study enabled an in-depth investigation of intra-individual variability of microbial and host immune function simultaneously. Intra-individual variability corresponded with microbial responses to both external perturbations of the gut environment, through the administration of antibiotics, and also to host immune functions. This large variability in preterm human infants makes it difficult to generalize results to a larger cohort, as other environmental factors (delivery mode, feeding type, hospital environment, etc.) can have non-negligent influences on the composition and functionality of gut microbiota and host. However, the findings in this study should drive interest in additional longitudinal studies with the recruitment of larger cohorts and a more balanced experimental design in terms of the timing of sample collection and control of environmental factors, including antibiotic administration.

Microbial mechanisms, such as antibiotic resistance, play a pivotal role in shaping the early-life preterm gut. The present study indicates that, in addition to antibiotic resistance mechanisms, metal homeostasis mechanisms correlate with community establishment dynamics. In fact, metal homeostasis may play a more prominent role in early-life community establishment than previously recognized. Metals are involved in many reactions and are essential to life. They are essential cofactors required for many reactions for both the host and microbes in the intestinal environment, and lack of these metals leads to organismal damage and death (Pajarillo et al., 2021). However, excess levels of metals also have negative impacts on cellular processes, such as the mismetallation of proteins, and high metal concentrations can be toxic due to their ability to disrupt normal metabolic functions and cause spontaneous redox cycling (Becker and Skaar, 2014). Organisms that have developed mechanisms that lead to resistance to host defenses involved in the trafficking of metals, based on either toxicity or starvation, have a better chance of survival and establishment in the gut. These early-life community dynamics play critical roles in developing the host immune system and ultimately host health through the interactions between microbiota and the host immune proteins.

Among metal trafficking activities, the interplay between host immune responses and gut microbiota for the utilization of copper and iron appears to play a vital role in community assembly for some infants in this cohort. While copper is essential for biological processes, excess intracellular copper is toxic because of its potential to mismetallate proteins since it has a higher affinity for noncognate ligands due to its location in the Irving-Williams series, which allows it to disrupt the binding of other transition metals like iron, manganese, and zinc (Casanova-Hampton et al., 2021). Previous studies of bacterial isolates have demonstrated that copper toxicity targets iron–sulfur-containing proteins *via* iron displacement from solvent-exposed iron–sulfur clusters (Macomber and Imlay, 2009; Ding et al., 2011; Djoko and McEwan, 2013). Organisms have developed several strategies based on the trafficking of both copper and iron to deal with host-imposed copper toxicity.

Based on the observed protein expression in infant 61, Figure 4B is a simplified representation of interactions between the host immune response and *E. cloacae* in this infant. Following phagocytosis, inflammatory agents, such as lipopolysaccharide stimulate copper uptake in macrophages by inducing the expression of copper importers at the plasma membrane. Cytoplasmic Cu (I) is then delivered to copper pumps which undergoes trafficking to the phagolysosome compartment where copper ions are loaded. Accumulation of Cu (I) within the phagolysosome contributes to bacterial killing through membrane damage, displacement of iron by copper in iron–sulfur (Fe-S) clusters, and formation of reactive oxygen species (Festa and Thiele, 2012; Koh and Henderson, 2015). In the bacterial cytoplasm, sensing of cytoplasmic Cu (I) by CueR induces the expression of copper transporter CopA, which effluxes Cu (I) into the periplasm. It is then transformed into the less toxic form of Cu (II) by the multicopper oxidase CueO or exported out of the cell. Both CopA and CopB are copper P-type ATPases; however, the exact physiological function of CopB has not been determined (Solioz, 2019). CopA has a low Cu (I) affinity and high turnover rate and is involved in exporting excess Cu (I) into the periplasm. CopB has a high Cu (I) affinity and low turnover rate and is suggested to transport Cu (I) into the periplasm for subsequent insertion into cuproenzymes) (Andrei et al., 2020). To complement strategies to export excess Cu (I) out of the cell or transform it to a usable form, previous work has demonstrated that some microbes have developed tactics to oxidize siderophores as a mechanism to deal with high levels of intracellular copper (Grass et al., 2004). In the presence of copper, the multicopper oxidase CueO oxidizes enterobactin, which circumvents enterobactin-mediated reduction of Cu (II) to Cu (I), and the resulting oxidation product, 2-carboxymuconate, sequesters copper. The oxidation process also releases chelated iron from the siderophores, maintaining the proper balance of copper and iron concentrations in the periplasm, which helps prevent mismetallation and damage to the cell. Studies have shown that *E. coli* lacking or defective in enterobactin biosynthesis and ferric-enterobactin uptake are more sensitive to copper toxicity (Gosalbes et al., 2016; Casanova-Hampton et al., 2021).

While there are many similarities in the usage of copper and iron by bacteria, the host has developed very different mechanisms to utilize these transition metals to restrict bacterial growth through either toxicity or limitation. During bacterial infection, phagocytic cells accumulate Cu (I) in cytoplasmic vesicles that partially fuse with the phagolysosome, flooding invading microbes with toxic levels of copper ions (Festa and Thiele, 2012). The accumulation of Cu (I) in the phagolysosome may depend upon the trafficking of ATP7A to the membranes of these vesicles (Fu et al., 2014). ATP7A is a Cu-ATPase that transports cytosolic copper (Cu2+) to the phagosomal lumen. Previous work of silencing ATP7A expression in mouse macrophages attenuated bacterial killing, which suggests a role for ATP7A-dependent copper transport in the bactericidal activity of macrophages (White et al., 2009). In addition, another study has shown that expression of ATP7A was downregulated in mouse colon tissue following antibiotic treatment, further supporting the idea that the presence of microbiota is essential in initiating this host immune response (Miller et al., 2019). PDZD11 and binding partner PLEKHA5 interact with ATP7A to influence its activity in cellular copper trafficking (Stephenson et al., 2005; Sluysmans et al., 2021). In total, six out of 17 infants expressed proteins related to copper trafficking for antimicrobial activities, including ATP7A, PDZD11, CTR1, and PLEKHA5. In this study, the increase in the expression of host proteins related to ATP7A corresponding to shifts in microbial organismal abundances that were not associated with external perturbations such as dietary changes or antibiotic treatment suggest that initiation of this host immune response was correlated to shifts in the abundance of these organisms. The host utilizes several mechanisms for maintaining iron homeostasis to limit pathogenic growth at mucosal interfaces through the restriction of metal-chelating proteins such as lactoferrin and lipocalin-2 (LCN2). As lactoferrin is a typical mass spectrometry contaminant (found in exocrine secretions such as sweat), this protein was removed from the analysis as the source of this protein could not be confirmed in the measurement. One proposed mechanism to enhance the host’s antimicrobial activities of copper toxicity is the increased expression of the inflammation-associated protein LCN2 to control the competition for iron during infection by binding and sequestering various siderophores produced by enteric bacteria (Clifton et al., 2019). In this study, LCN2 was present in 90 samples, and the expression of LCN2 by almost all infants in the study indicates an active attempt by the host to limit iron harvesting and ultimately bacterial growth by the microbes producing siderophores.

The longitudinal measurements of microbial functional dynamics in infant 61 indicate that following the increase in the apparent host-imposed copper toxicity on day eight, the relative abundance of the microbiota is driven by the ability to respond to this host immune response. In summary, the observed organismal abundance trends in this infant during periods of antibiotic administration and host immune-mediated metal toxicity show that microbial resistance and homeostasis mechanisms to both of these perturbations are critical for persistence and maintaining establishment in the developing gut. For example, lacking either antibiotic resistance mechanisms (*E. faecalis*) or copper efflux proteins (*K. pnuemoniae)* relates to a decrease in relative abundance, while expression of proteins related to both of these mechanisms (*E. cloacae*) relates to increased relative abundance in the community after environmental stress. Overall, the observation that a microbe’s ability to circumvent the host’s attempt to limit some microbial growth through copper-mediated toxicity and limitation of iron harvesting contributed to its persistence and establishment in the environment.

Several studies investigating the activity of calprotectin during bacterial infections have found that it can be detected in the feces of patients with other types of intestinal inflammation, such as colon cancer and inflammatory bowel disorders (Kehl-Fie et al., 2011; Diaz-Ochoa et al., 2016; Ho et al., 2019). Breastmilk contains high levels of calprotectin, and this heterodimer has been suggested to be involved in host immune regulation (Healy et al., 2022). In this cohort, we observed changes in fecal calprotectin levels that were not associated with changes in consumption of formula and breastmilk, supporting the idea that changes in calprotectin levels were due to an inflammatory response and not due to diet. During the periods of altered host expression of calprotectin, there was a corresponding pattern of increased manganese import in selected organisms in several infants based on the expression of proteins related to the import of this metal. Figure 5B illustrates how the host can impose manganese and zinc starvation, and one mechanism microbes might use to overcome this, as observed in infant 39 during the period of increased host expression of calprotectin. During the infection process, enterocytes are stimulated to express the cytokines, such as IL-17 and IL-22, which bind to receptors on neutrophils, thereby promoting transmigration of the neutrophils to the site of infection where they release calprotectin into the intestinal lumen where it binds to zinc and manganese, making these nutrients unavailable to the microbes (Liu et al., 2012).

Some microbes have overcome this strategy by utilizing additional membrane transporters such as the ones shown in the figure that enhance their ability to import manganese into the cell. Based on the presence of manganese import-related transporters and the expression of several manganese-dependent proteins in microbes who increased in abundance during periods of high host expression of calprotectin, we can infer host sequestration of this metal did not impact the growth of *L. rhamnosus* in infant 39. This enables *L. rhamnosus*, and other microbes with these enhanced manganese and zinc import capabilities, to outcompete other microbes lacking these mechanisms (*C. freundii)* in manganese and zinc-limited environments.

As both manganese and zinc are cofactors for many enzymes involved in critical processes required for bacterial growth, including central metabolism, mechanisms for bacteria to compete with the host for these metals are essential for the survival of the organisms (Becker and Skaar, 2014). Both manganese and zinc are also cofactors for several proteins directly involved in microbial response to other innate immune responses and to antibiotic intervention for the treatment of infection. Specifically, enteric pathogens require manganese as a cofactor for superoxide dismutase to protect the cell from host-imposed oxidative stress (Kehl-Fie et al., 2011). Interestingly, zinc is a necessary cofactor for metallo-beta-lactamases, which are capable of inactivating beta-lactam antibiotics (Kim et al., 2013). This may explain why some of the organisms in this study that expressed both the relevant antibiotic resistance-related proteins and manganese import proteins increased in relative abundance during periods of antibiotic administration in contrast to organisms with similar antibiotic resistance capabilities that lacked manganese importers, which decreased in abundance. In general, as both manganese and zinc are attractive targets for the host to limit as part of an immune response, microbial homeostasis mechanisms for these metals may play a more significant role in community assembly in the preterm infant gut than previously recognized.

Metaproteomics studies provide a unique opportunity to study microbial colonization, establishment, and persistence in an *in vivo* clinical context, as this approach allows simultaneous measurement of both host and microbial function. In this study, community assembly and persistence of microbiota in the early-life developing preterm gut were correlated with individual microbial responses to antibiotic administration and host-imposed metal bactericidal control. Specifically, microbial protein expression of mechanisms related to antibiotic resistance and metal homeostasis were predominant in community members that persisted in the highly dynamic gut environment over the first several weeks of life. A deeper understanding of the functional interplay at the host-microbe interface may clarify the potential immunomodulatory and defense roles of microbiota that are relevant to dysbiotic conditions, such as necrotizing enterocolitis, in premature infants.

## Materials and methods

### Sample selection, preparation, and measurement

Sample collection, processing, and metaproteomic measurements by LC–MS/MS were already described (Xiong et al., 2017; Brown et al., 2018). Briefly, using necrotizing enterocolitis (NEC) as a representative dysbiotic condition, 91 metaproteomic measurements were taken from 17 preterm infant fecal samples over the first 90 days of life. Six infants developed NEC during the study (Supplemental Table 3). 0.3 g of raw fecal stool was processed using an indirect enrichment strategy (Xiong et al., 2015)^,^ and 50ug of digested peptides were analyzed in technical duplicate *via* two-dimensional nanospray liquid chromatography–tandem mass spectrometry (LC–MS/MS) on an LTQ-Orbitrap Elite mass spectrometer (Thermo Scientific).

### Database searching and construction of protein databases

Matched metagenome-derived protein databases for each sample were compiled into infant-specific databases containing any predicted proteins for that individual. Metagenomic data used for the construction of the predicted protein databases used in the present study was conducted previously (Brown et al., 2018). In addition, the databases contained protein sequences from the human reference proteome (UniProtKB/TrEMBL, UP000005640), common mass spectrometry contaminants, and reverse sequences of all entries in the database to control the false discovery rate (FDR). Previously collected MS/MS spectra were searched using Proteome Discover 2.3 (Thermo Scientific), employing MSAmanda 2.0 and Elutator (Dorfer et al., 2018, 2021) with the second search feature in MSAmanda2.0 turned off. Peptide spectrum matches (PSMs) were required to be fully tryptic with up to two miscleavages, a static carbamidomethylation modification of 57.0214 Da for cysteine residues, and a dynamic oxidation modification of 15.9949 Da for methionine residues. FDR was assessed by matches to reverse decoy sequences and controlled at 1% on the peptide level. To alleviate the ambiguity associated with shared peptides, proteins were clustered into protein groups by 100% identity for microbial proteins and 85% amino acid sequence identity for human proteins using UCLUST (Edgar, 2010). FDR-controlled peptides were then quantified according to the chromatographic area under the curve (AUC). Peptide AUC values obtained through match-between-runs (MBR) were included for each sample if there was one MS2 event per sample group (infant). Peptide intensities were summed to estimate protein-level abundance based on peptides that uniquely mapped to one protein group. Technical replicates were merged at the protein level after protein roll-up. One of the files for infant 65 (DOL 17) was not searchable, so only the other replicate was included in the analysis. Protein abundances from all 91 samples were log-transformed, normalized at the sample level by LOESS, and standardized across the entire dataset by median absolute deviation (MAD) and median centering. Missing values were imputed to simulate the mass spectrometer’s limit of detection with a downshift of 2.4 and a width of 0.3.

### Functional annotation of proteins

Annotation of immune-related proteins was performed using the Reactome Database (version 77). Proteins that mapped to several immune pathways were counted for each pathway. Further analysis was performed at the pathway level with the requirement that at least one protein in the pathway is unique. For annotation of microbial proteins, taxonomy assignments were performed as previously described (Brown et al., 2018). KO terms were identified using the eggNOG database emapper function (version emapper-1.0.3-35-g63c274b) (Huerta-Cepas et al., 2019). All quantified microbial protein groups (Supplemental Table 1) were searched against the Comprehensive Antibiotic Resistance Database (CARD) (Alcock et al., 2020) to identify antibiotic resistance-related proteins. Protein sequence hits to the database that mapped to more than one antibiotic resistance ortholog (ARO) were counted for each matching ARO. Both perfect and strict hits were included in the analysis.

### Data analysis

The samples’ dissimilarities were assessed using Jaccard and Bray-Curtis distances for NMDS ordinations using the metaMDS () function in the vegan R package. Trends in functional dissimilarity between samples were assessed using a linear mixed-effects model (LMM) with the lmer function in the lme4 package in R. Fixed effects analyzed included day of life, gestational age, birth age, delivery mode, feeding type, NEC diagnosis, infection diagnosis, sepsis diagnosis, and birth weight. Infant ID was included as a random effect in the model to account for repeated measures over time. Before the Jaccard distance calculations, the microbial protein groups were summed to the KO term level. Permutational multivariate analysis of variance (PERMANOVA), applying 999 permutations, was used to assess statistical significance in beta diversity between categorical variables of interest—related to patient health metadata, including delivery mode, antibiotic usage, health/disease status, etc. The betadisper package in R was used as a complementary assessment to the PERMANOVA testing to test for homogeneity of multivariate dispersion to ensure the within-group dispersion was not significant using 999 permutations. A category was considered to be significant in the analysis if there was a significant PERMANOVA result (value of *p* <0.05) and an insignificant betadisper result (value of *p*>0.05) after Benjamini-Hochberg correction.

For the relative organismal abundance alluvial plots, the “other” category all proteins with no genus or species level taxonomy classification or if the total organismal abundance was less than 1% of total abundance for any timepoint in the infant.

## Data availability statement

Protein sequence databases and metaproteomic data with relevant metadata are available at https://massive.ucsd.edu/ under accession number MSV000086096.

The sequencing read datasets used to generate the protein databases have been previously deposited in the NCBI sequence read archive and are available under BioProjects PRJNA294605 and PRJNA376566.

## Ethics statement

The studies involving human participants were reviewed and approved by University of Pittsburgh IRB committee. The patients/participants provided their written informed consent to participate in this study.

## Author contributions

RH and SP conceived the study. SP analyzed the data and wrote the manuscript. MM and RH acquired funding and provided administration for the project. RH supervised the study. All authors read, edited, and provided feedback on the manuscript. All authors contributed to the article and approved the submitted version.

## Funding

This work was funded by the NIH R01GM103600. SP was supported by an Oak Ridge Innovation Institute’s EERE Workforce Development project fellowship.

## Licenses and Permissions

This manuscript has been authored by UT-Battelle, LLC under Contract No. DE-AC05-00OR22725 with the U.S. Department of Energy. The United States Government retains and the publisher, by accepting the article for publication, acknowledges that the United States Government retains a non-exclusive, paid-up, irrevocable, world-wide license to publish or reproduce the published form of this manuscript, or allow others to do so, for United States Government purposes. The Department of Energy will provide public access to these results of federally sponsored research in accordance with the DOE Public Access Plan (http://energy.gov/downloads/doe-public-access-plan).

## Conflict of interest

The authors declare that the research was conducted in the absence of any commercial or financial relationships that could be construed as a potential conflict of interest.

## Publisher’s note

All claims expressed in this article are solely those of the authors and do not necessarily represent those of their affiliated organizations, or those of the publisher, the editors and the reviewers. Any product that may be evaluated in this article, or claim that may be made by its manufacturer, is not guaranteed or endorsed by the publisher.
