# Supplementary material for: Antibiotic resistance and host immune system-induced metal bactericidal control are key factors for microbial persistence in the developing human preterm infant gut microbiome
Source: Front Microbiol. 2022 Nov 21;13:958638. doi: 10.3389/fmicb.2022.958638 (PMC9720133; doi:10.3389/fmicb.2022.958638)
Supplement: Supplementary file 5 [file Data_Sheet_1.PDF]

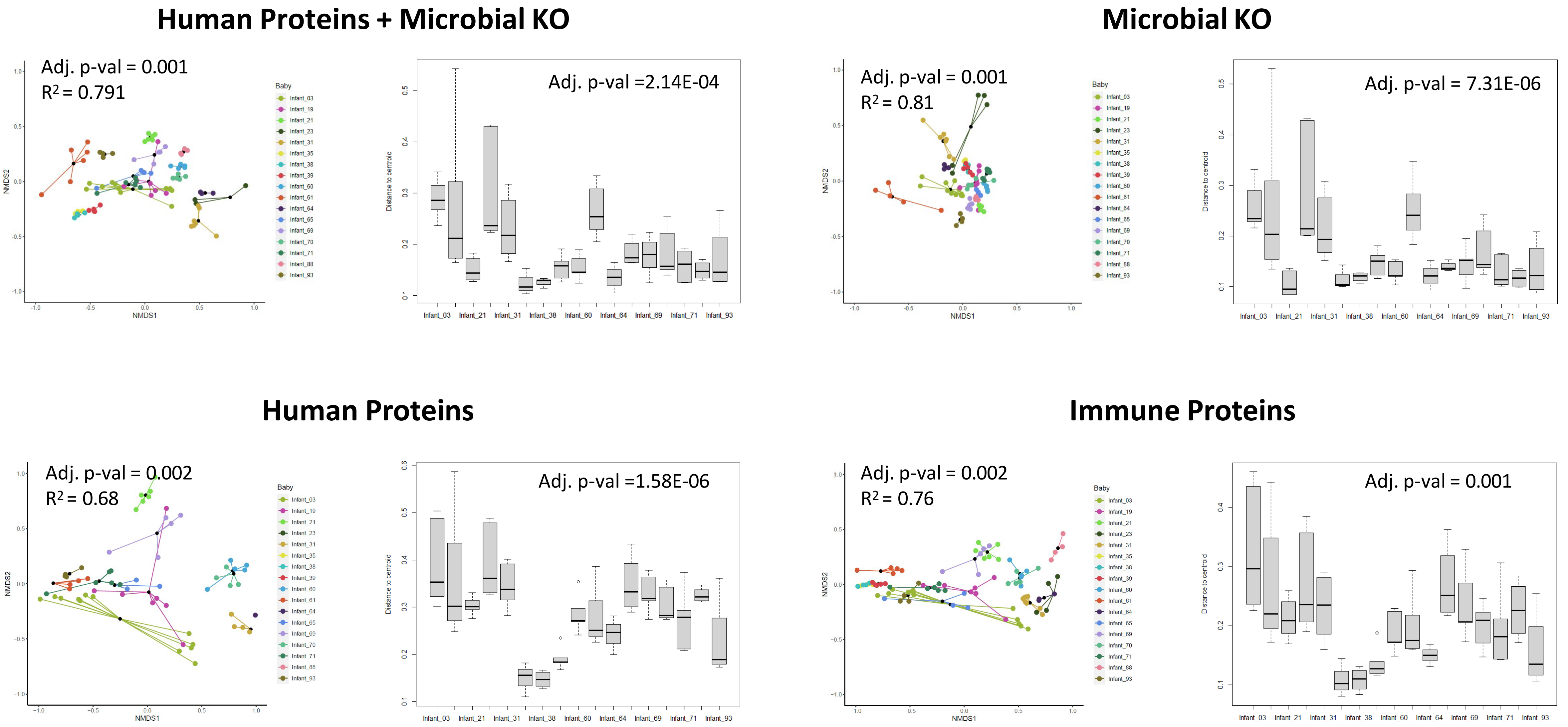

Supplemental Figure 1. Functional β-diversity by proteins source for the 91 samples in the study based on the infant source of the samples. Non-metric multidimensional scaling (NMDS) of Jaccard distances for the collective functionality (based on presence or absence of proteins or KEGG ortholog groups [KOs]) of each sample for **(A)** Human immune proteins and microbial KOs, **(B)** microbial KOs, **(C)** human proteins, **(D)** human immune proteins. Boxplots showing the dispersion of these distances, as assessed with the *betadisper* function of the vegan package.

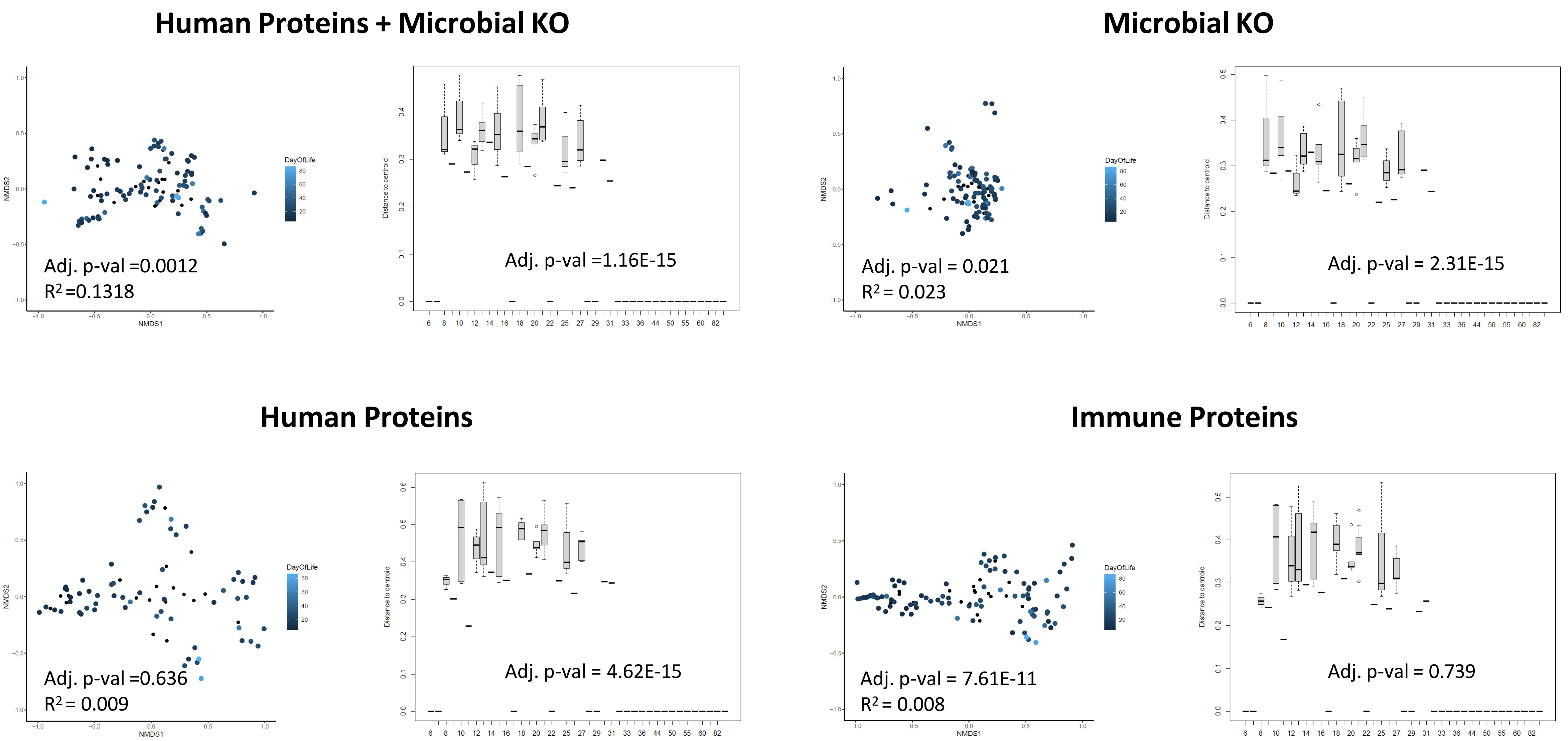

Supplemental Figure 2. Functional β-diversity by proteins source for the 91 samples in the study based on the age of the infant during sample collection (day of life). Non-metric multidimensional scaling (NMDS) of Jaccard distances for the collective functionality (based on presence or absence of proteins or KEGG ortholog groups [KOs]) of each sample for **(A)** Human immune proteins and microbial KOs, **(B)** microbial KOs, **(C)** human proteins, **(D)** human immune proteins. Boxplots showing the dispersion of these distances, as assessed with the *betadisper* function of the vegan package.

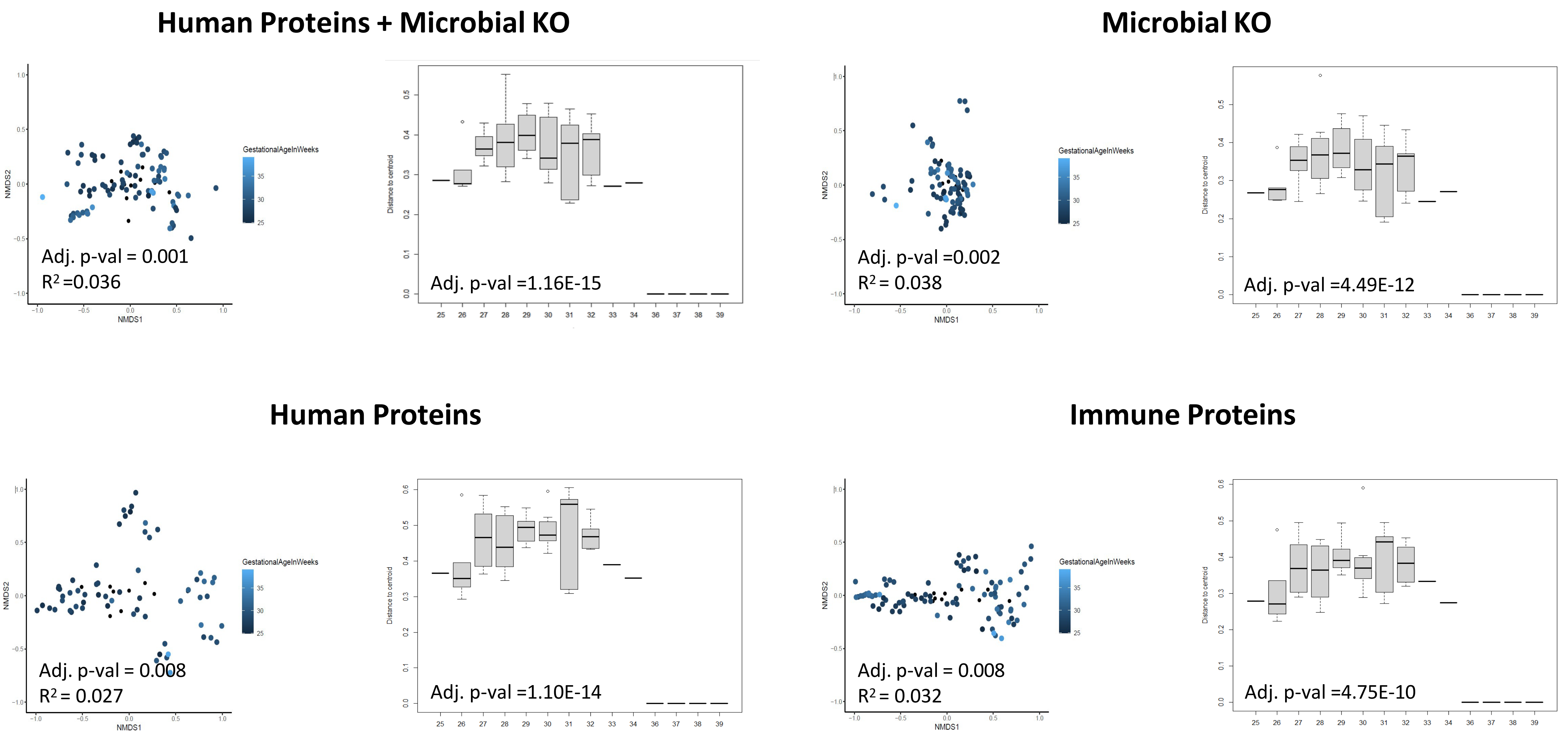

Supplemental Figure 3. Functional β-diversity by proteins source for the 91 samples in the study based on the age of the infant during sample collection (gestational weeks). Non-metric multidimensional scaling (NMDS) of Jaccard distances for the collective functionality (based on presence or absence of proteins or KEGG ortholog groups [KOs]) of each sample for **(A)** Human immune proteins and microbial KOs, **(B)** microbial KOs, **(C)** human proteins, **(D)** human immune proteins. Boxplots showing the dispersion of these distances, as assessed with the *betadisper* function of the vegan package.

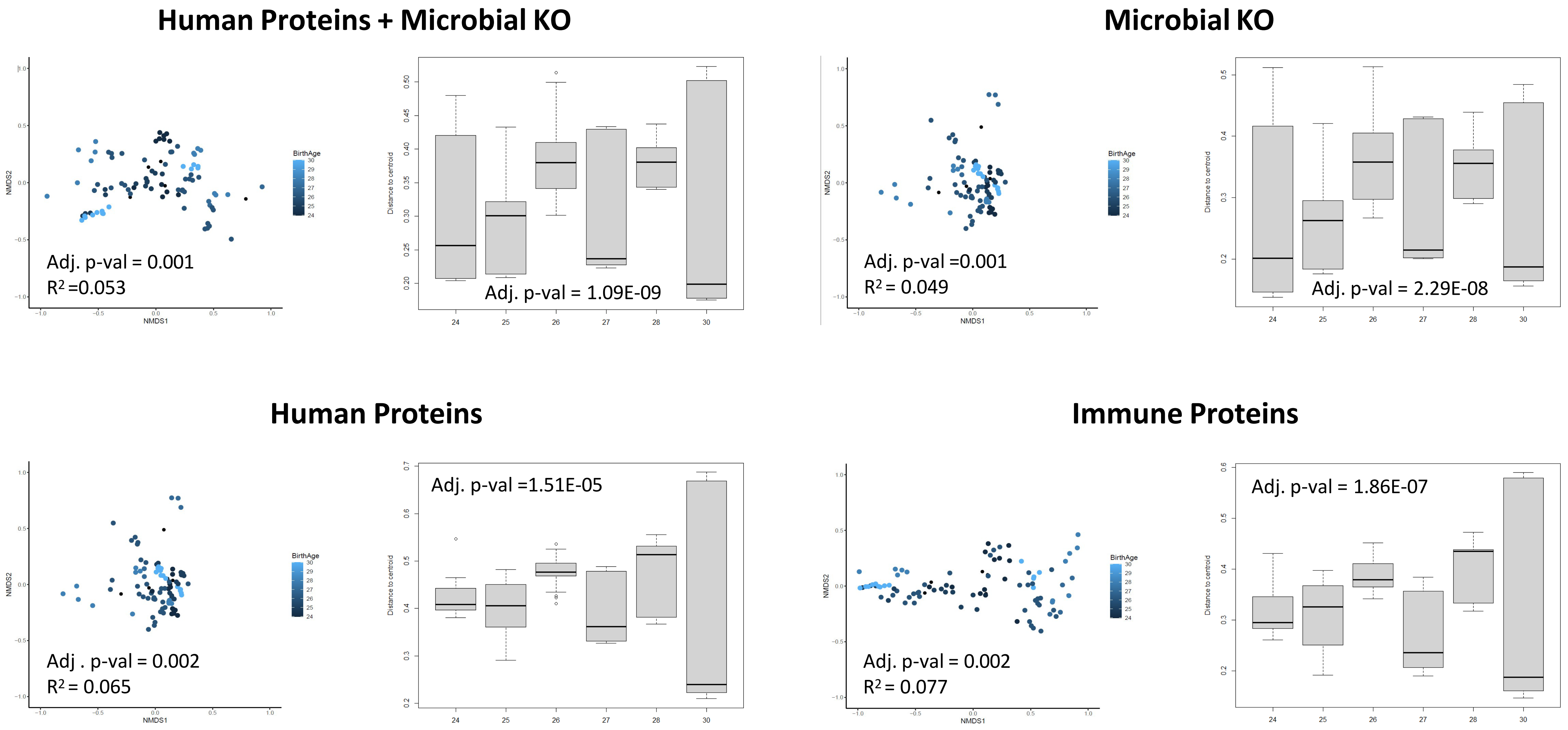

Supplemental Figure 4. Functional β-diversity by proteins source for the 91 samples in the study based on the birth age of the infant (gestational weeks). Non-metric multidimensional scaling (NMDS) of Jaccard distances for the collective functionality (based on presence or absence of proteins or KEGG ortholog groups [KOs]) of each sample for **(A)** Human immune proteins and microbial KOs, **(B)** microbial KOs, **(C)** human proteins, **(D)** human immune proteins. Boxplots showing the dispersion of these distances, as assessed with the *betadisper* function of the vegan package.

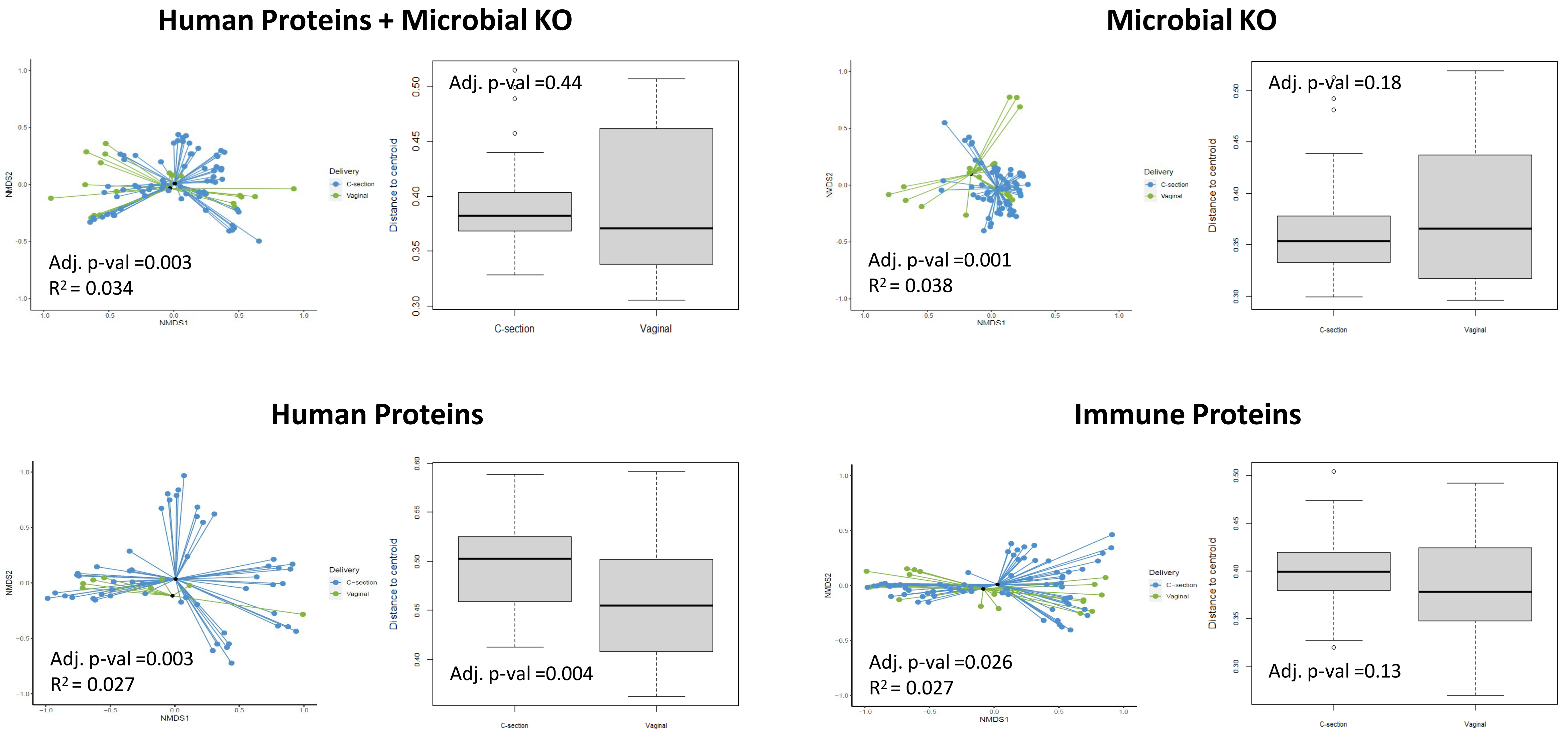

Supplemental Figure 5. Functional β-diversity by proteins source for the 91 samples in the study based on the delivery mode of the infant. Non-metric multidimensional scaling (NMDS) of Jaccard distances for the collective functionality (based on presence or absence of proteins or KEGG ortholog groups [KOs]) of each sample for **(A)** Human immune proteins and microbial KOs, **(B)** microbial KOs, **(C)** human proteins, **(D)** human immune proteins. Boxplots showing the dispersion of these distances, as assessed with the *betadisper* function of the vegan package.

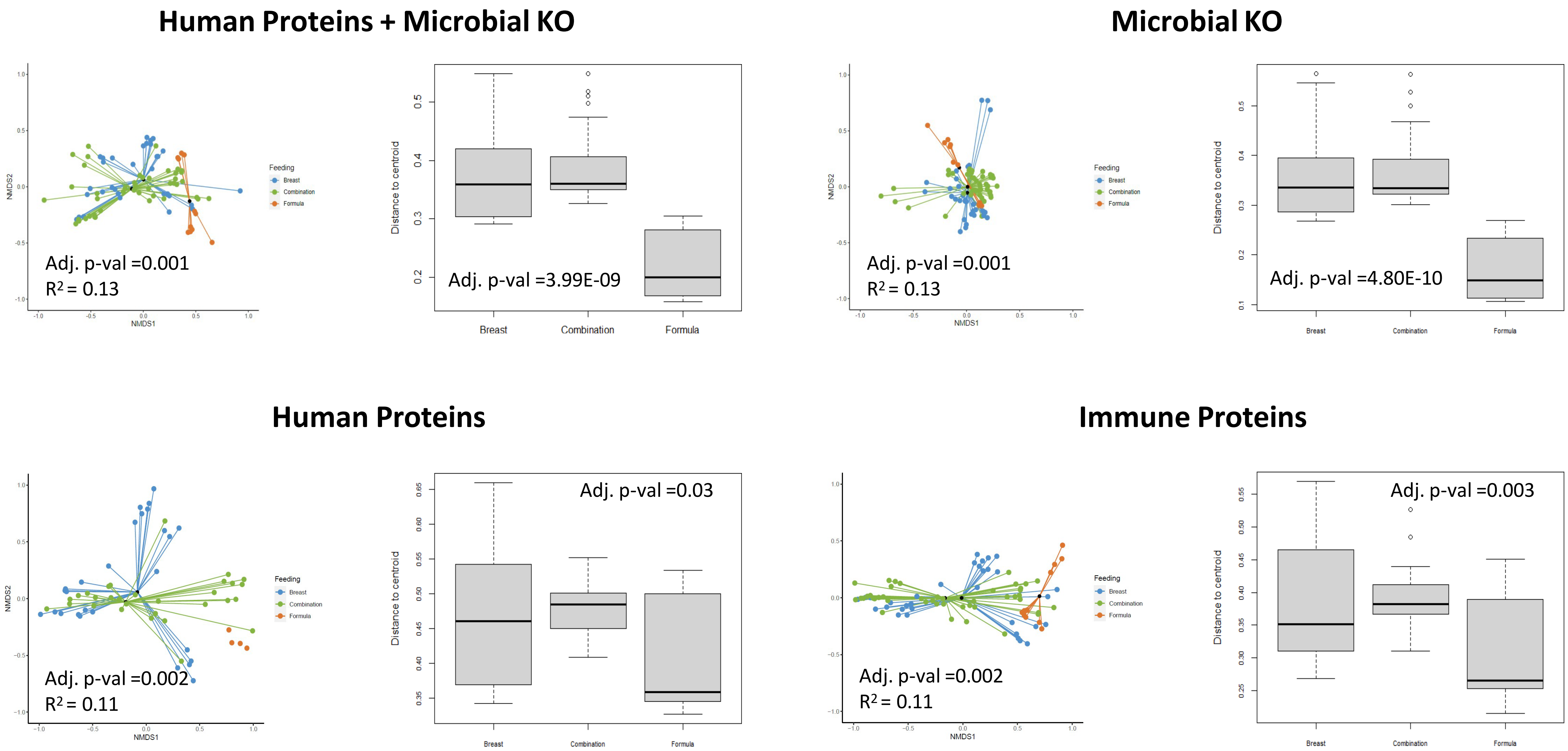

Supplemental Figure 6. Functional β-diversity by proteins source for the 91 samples in the study based on the feeding type of the infant at the time of sample collection. Non-metric multidimensional scaling (NMDS) of Jaccard distances for the collective functionality (based on presence or absence of proteins or KEGG ortholog groups [KOs]) of each sample for **(A)** Human immune proteins and microbial KOs, **(B)** microbial KOs, **(C)** human proteins, **(D)** human immune proteins. Boxplots showing the dispersion of these distances, as assessed with the *betadisper* function of the vegan package.

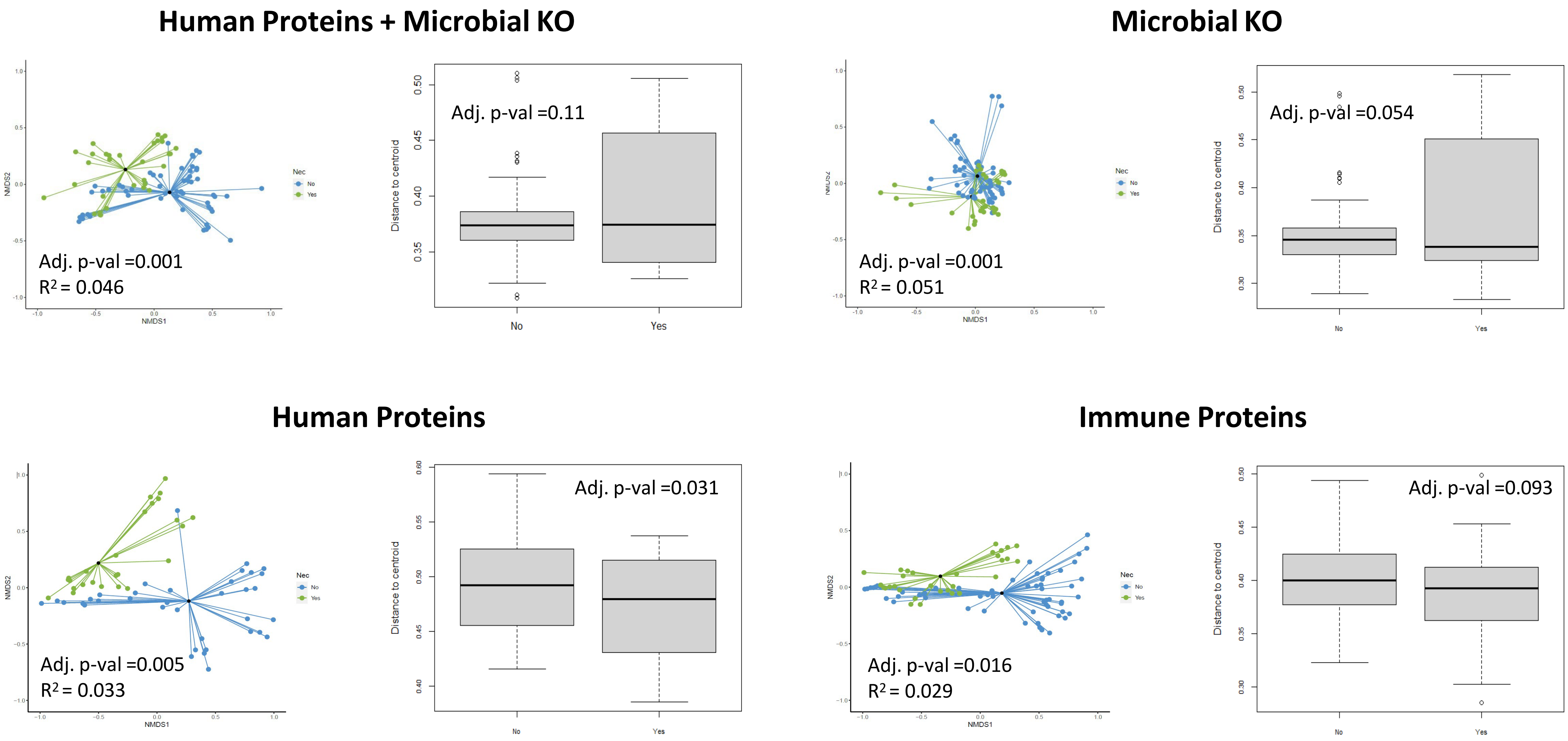

Supplemental Figure 7. Functional β-diversity by proteins source for the 91 samples in the study based on the diagnosis of necrotizing enterocolitis (NEC) for each infant during the course of the study. Non-metric multidimensional scaling (NMDS) of Jaccard distances for the collective functionality (based on presence or absence of proteins or KEGG ortholog groups [KOs]) of each sample for **(A)** Human immune proteins and microbial KOs, **(B)** microbial KOs, **(C)** human proteins, **(D)** human immune proteins. Boxplots showing the dispersion of these distances, as assessed with the *betadisper* function of the vegan package.

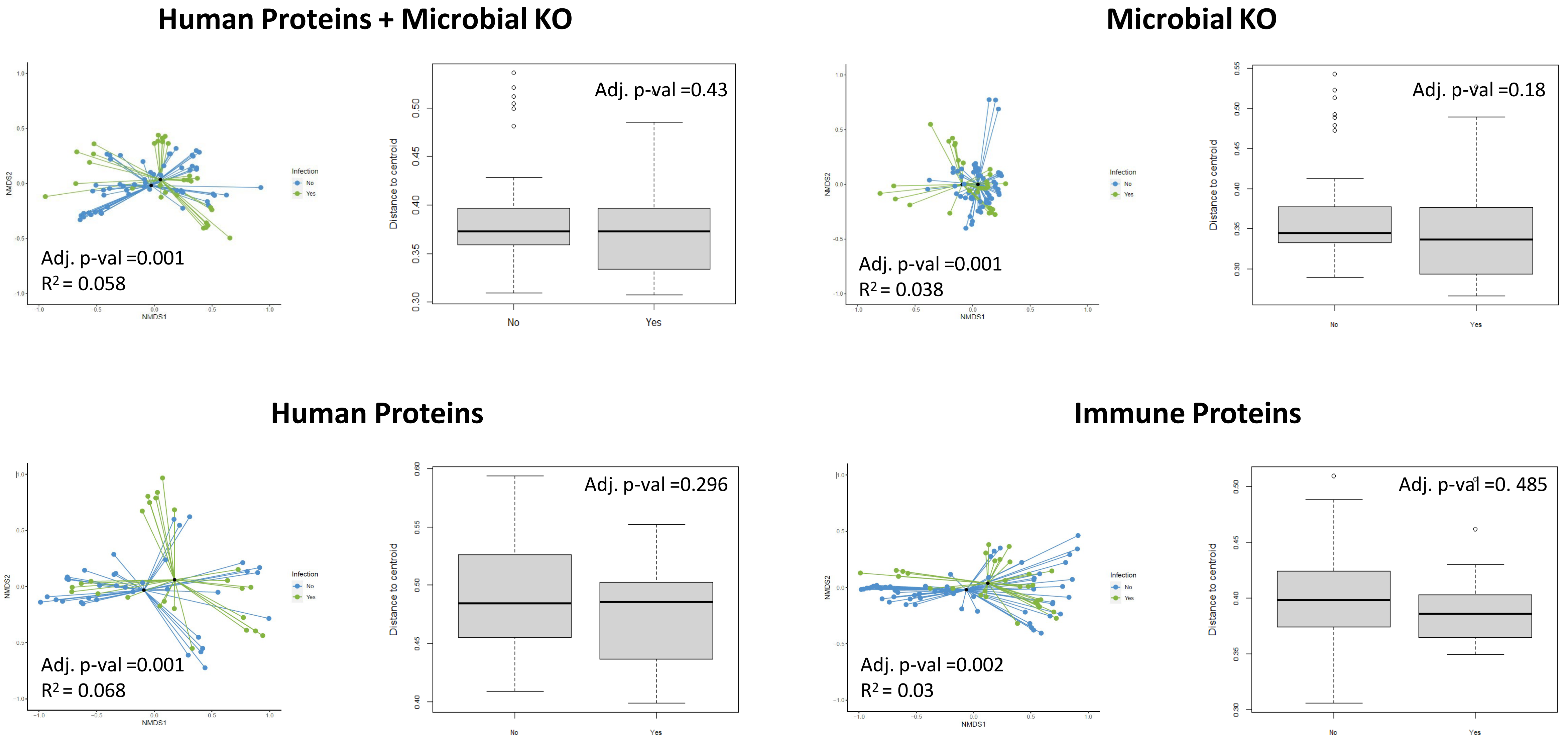

Supplemental Figure 8. Functional β-diversity by proteins source for the 91 samples in the study based on the diagnosis of infection for each infant during the course of the study. Non-metric multidimensional scaling (NMDS) of Jaccard distances for the collective functionality (based on presence or absence of proteins or KEGG ortholog groups [KOs]) of each sample for **(A)** Human immune proteins and microbial KOs, **(B)** microbial KOs, **(C)** human proteins, **(D)** human immune proteins. Boxplots showing the dispersion of these distances, as assessed with the *betadisper* function of the vegan package.

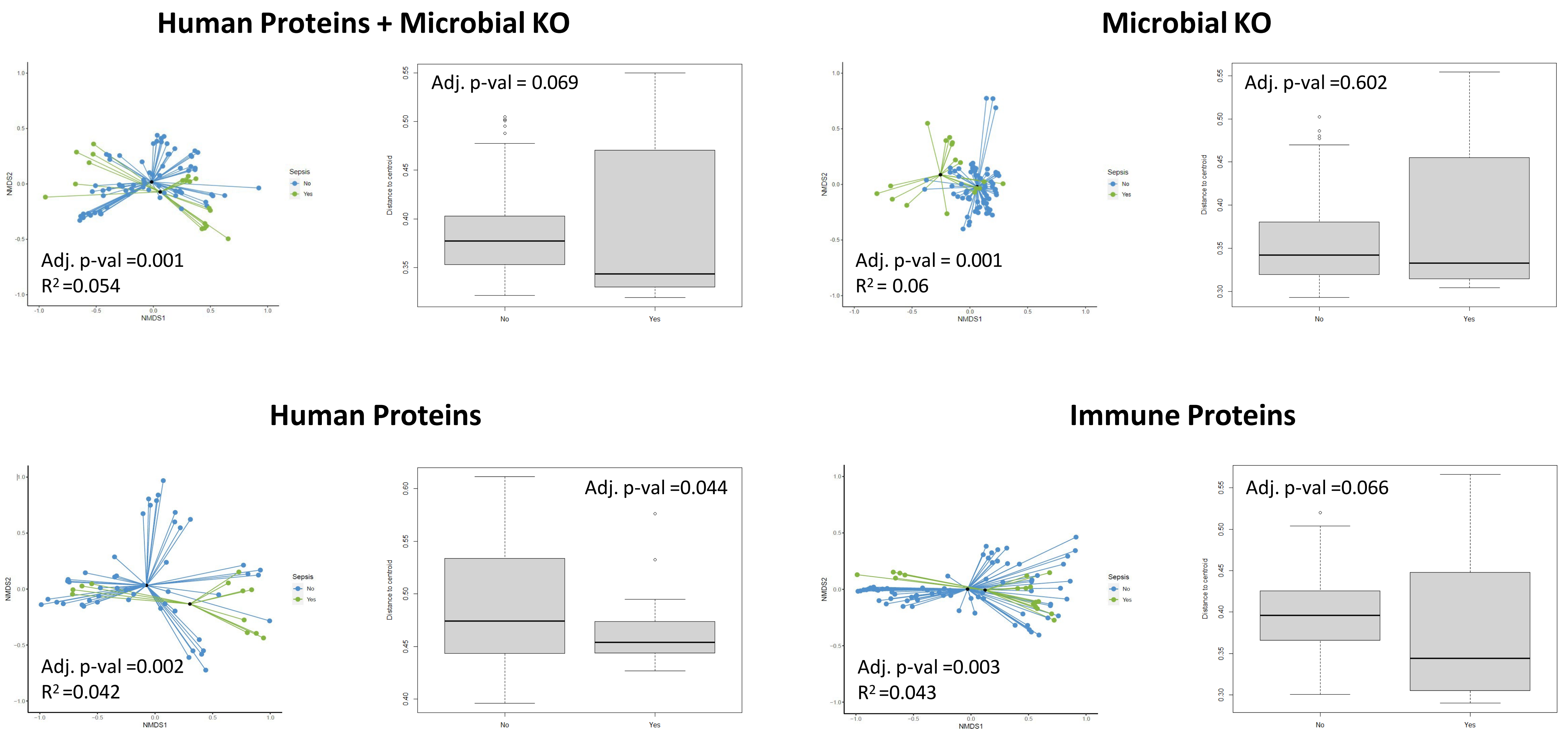

Supplemental Figure 9. Functional β-diversity by proteins source for the 91 samples in the study based on the diagnosis of sepsis for each infant during the course of the study. Non-metric multidimensional scaling (NMDS) of Jaccard distances for the collective functionality (based on presence or absence of proteins or KEGG ortholog groups [KOs]) of each sample for **(A)** Human immune proteins and microbial KOs, **(B)** microbial KOs, **(C)** human proteins, **(D)** human immune proteins. Boxplots showing the dispersion of these distances, as assessed with the *betadisper* function of the vegan package.

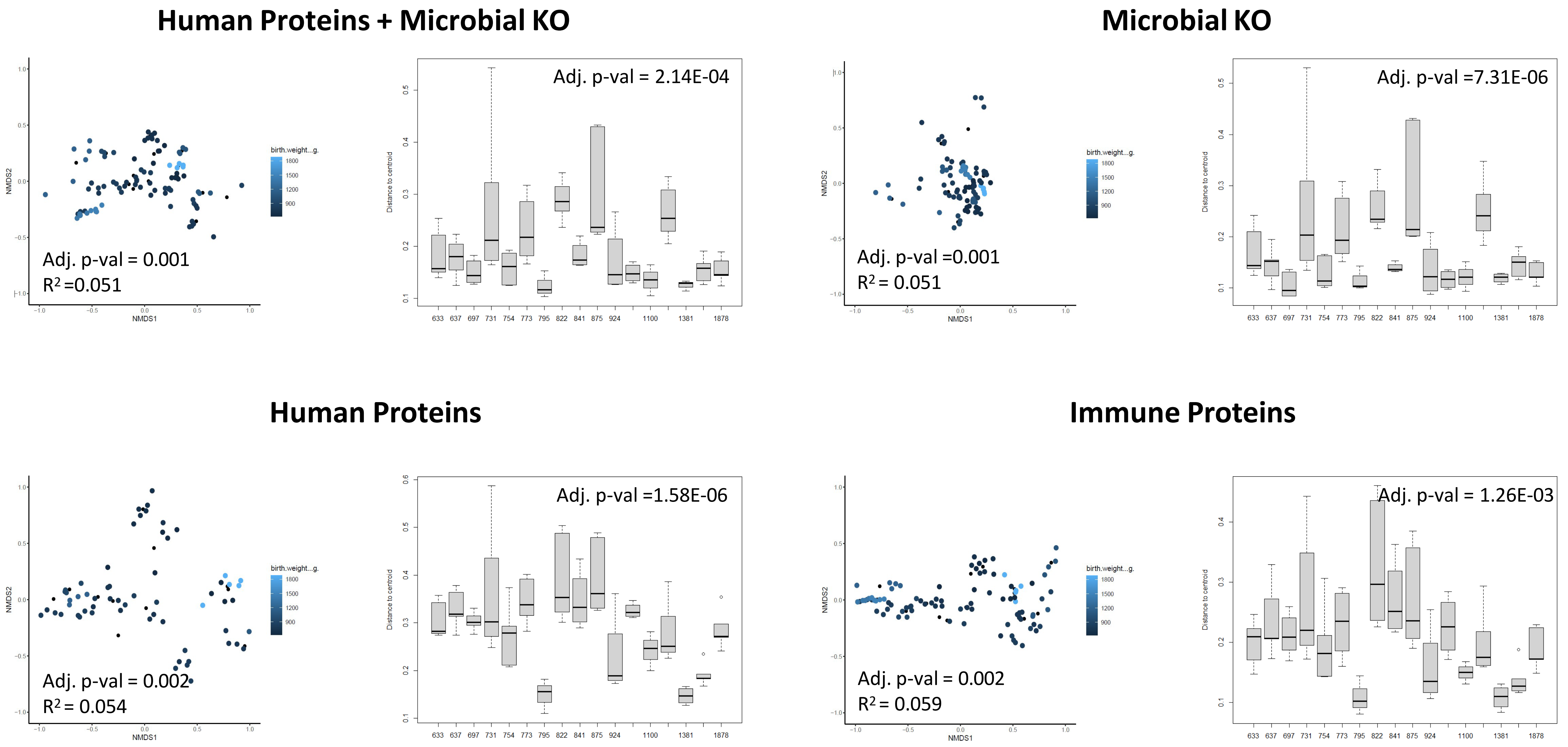

Supplemental Figure 10. Functional β-diversity by proteins source for the 91 samples in the study based on birth weight (g) of each infant. Non-metric multidimensional scaling (NMDS) of Jaccard distances for the collective functionality (based on presence or absence of proteins or KEGG ortholog groups [KOs]) of each sample for **(A)** Human immune proteins and microbial KOs, **(B)** microbial KOs, **(C)** human proteins, **(D)** human immune proteins. Boxplots showing the dispersion of these distances, as assessed with the *betadisper* function of the vegan package.

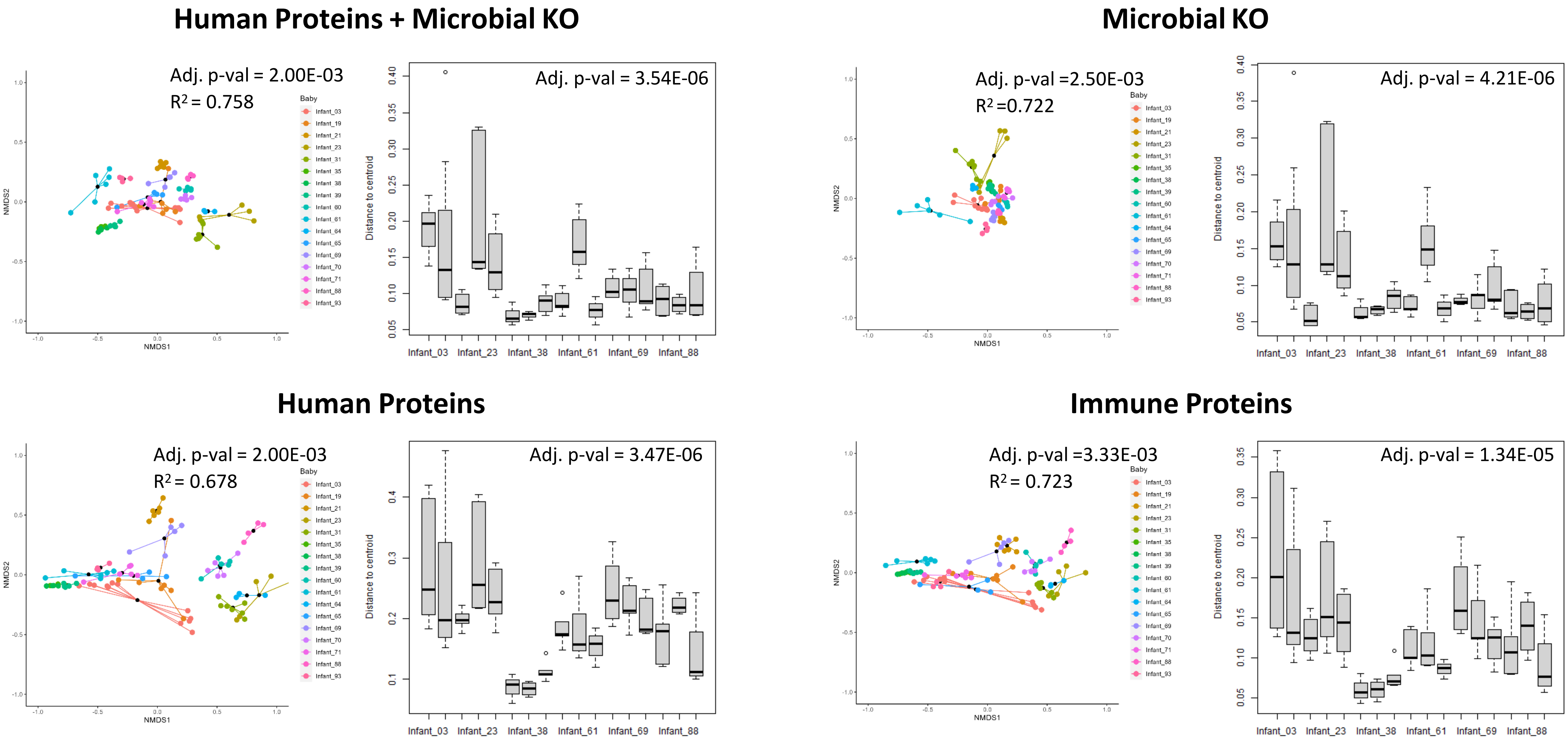

Supplemental Figure 11. Functional β-diversity by proteins source for the 91 samples in the study based on the infant source of the samples. Non-metric multidimensional scaling (NMDS) of Bray–Curtis distances for the collective functionality (based on presence/absence and abundance of proteins or KEGG ortholog groups [KOs]) of each sample for **(A)** Human immune proteins and microbial KOs, **(B)** microbial KOs, **(C)** human proteins, **(D)** human immune proteins. Boxplots showing the dispersion of these distances, as assessed with the *betadisper* function of the vegan package.

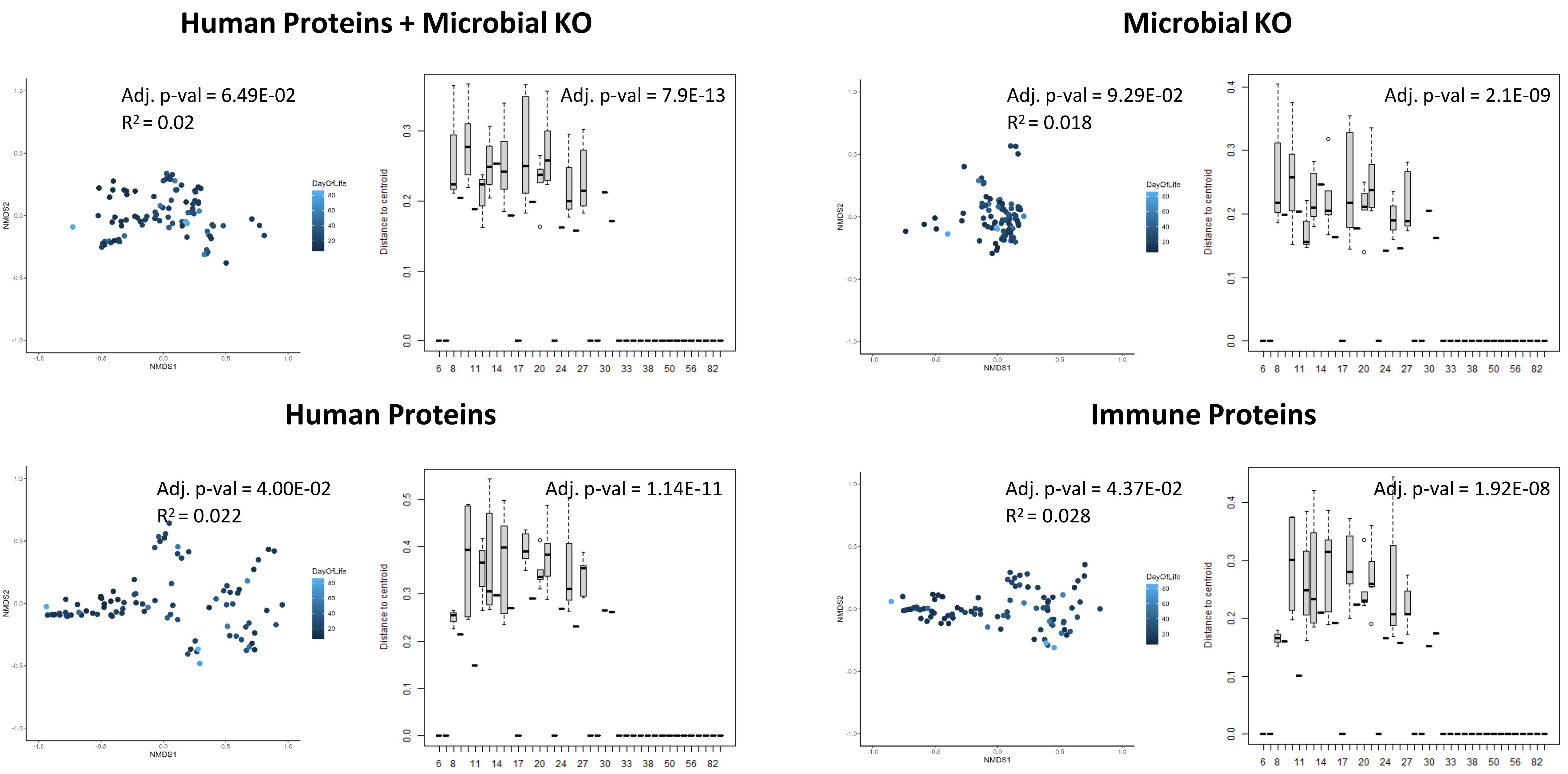

Supplemental Figure 12. Functional β-diversity by proteins source for the 91 samples in the study based on the age of the infant during sample collection (day of life). Non-metric multidimensional scaling (NMDS) of Bray–Curtis distances for the collective functionality (based on presence/absence and abundance of proteins or KEGG ortholog groups [KOs]) of each sample for **(A)** Human immune proteins and microbial KOs, **(B)** microbial KOs, **(C)** human proteins, **(D)** human immune proteins. Boxplots showing the dispersion of these distances, as assessed with the *betadisper* function of the vegan package.

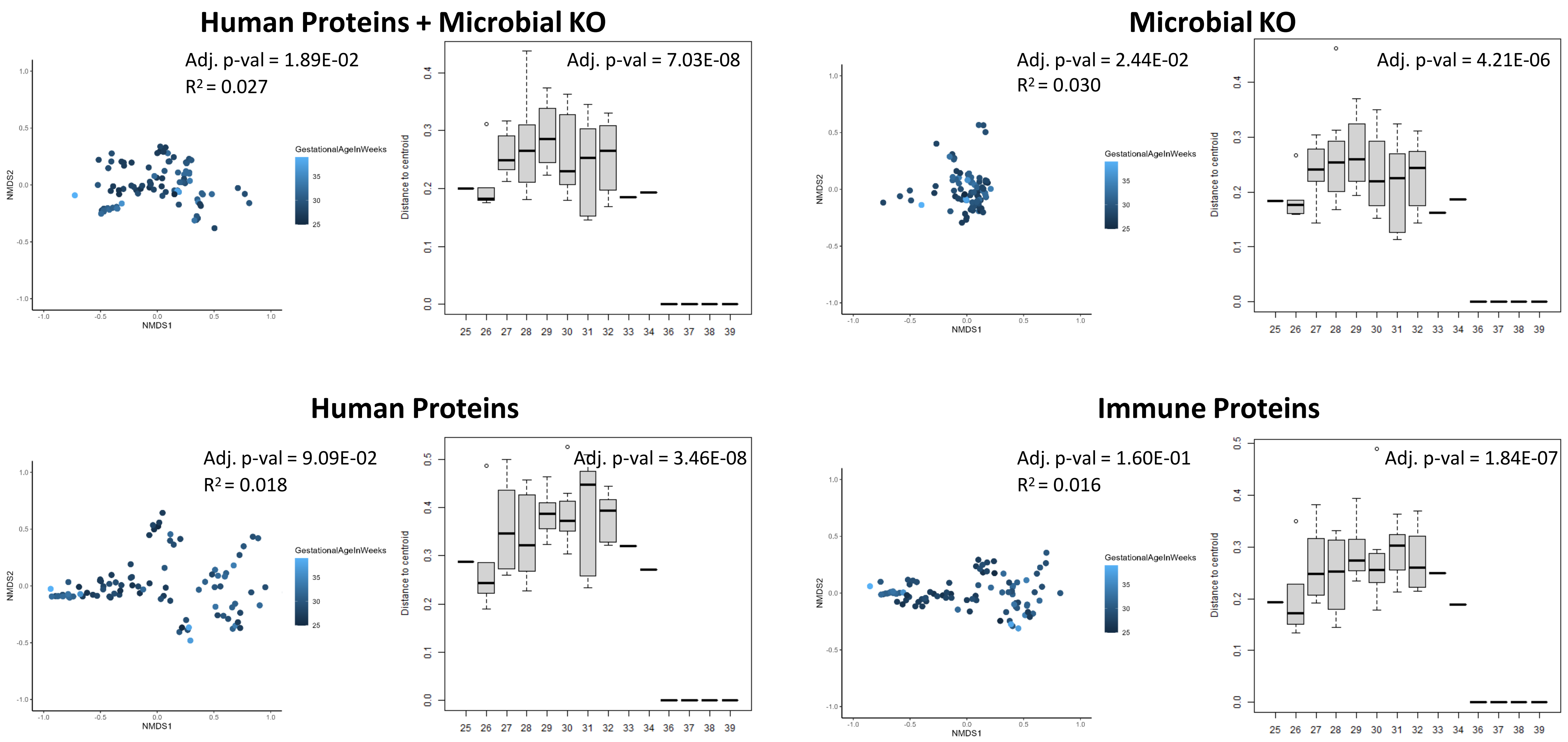

Supplemental Figure 13. Functional β-diversity by proteins source for the 91 samples in the study based on the age of the infant during sample collection (gestational weeks). Non-metric multidimensional scaling (NMDS) of Bray–Curtis distances for the collective functionality (based on presence/absence and abundance of proteins or KEGG ortholog groups [KOs]) of each sample for **(A)** Human immune proteins and microbial KOs, **(B)** microbial KOs, **(C)** human proteins, **(D)** human immune proteins. Boxplots showing the dispersion of these distances, as assessed with the *betadisper* function of the vegan package.

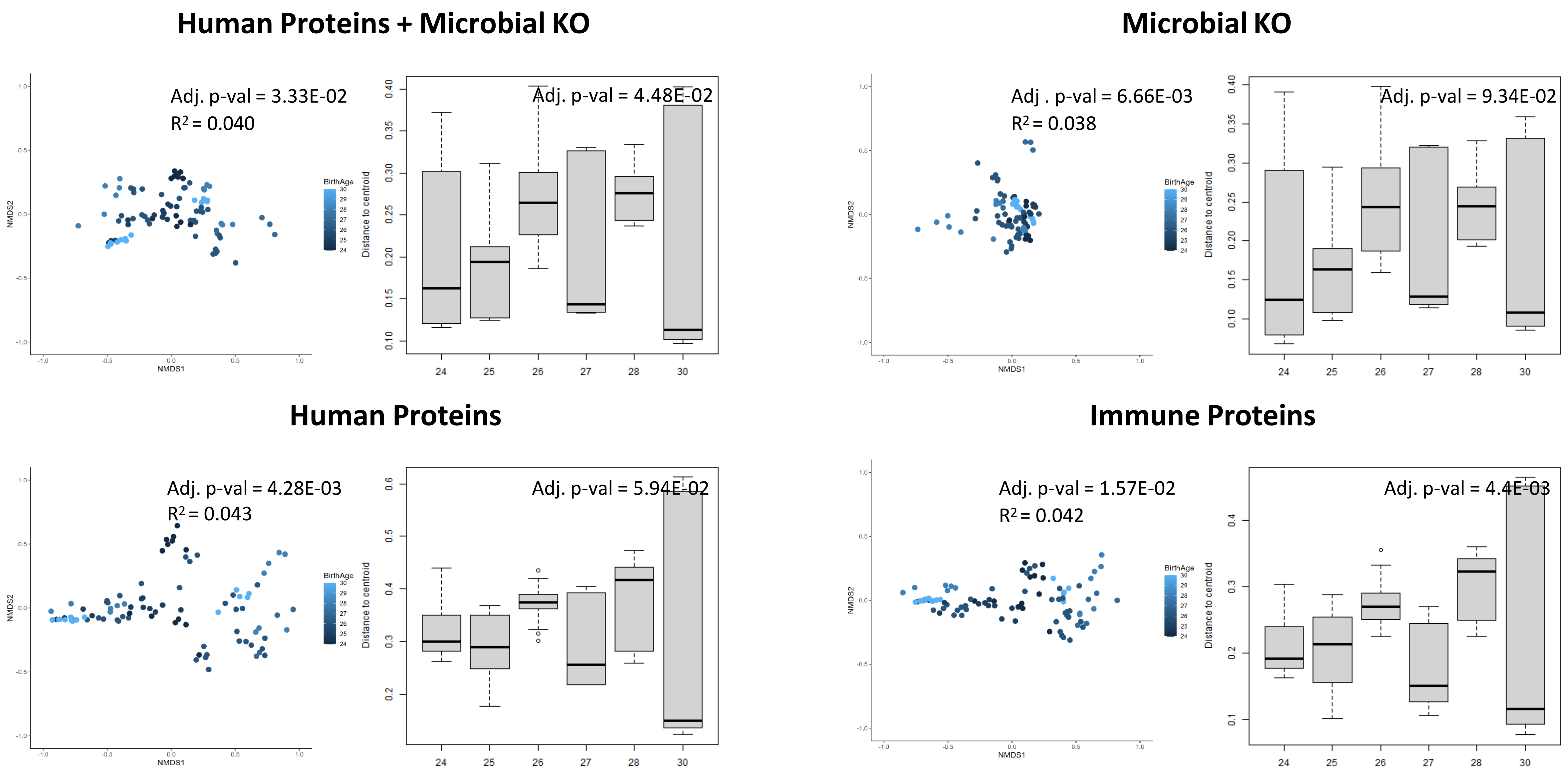

Supplemental Figure 14. Functional β-diversity by proteins source for the 91 samples in the study based on the birth age of the infant (gestational weeks). Non- metric multidimensional scaling (NMDS) of Bray–Curtis distances for the collective functionality (based on presence/absence and abundance of proteins or KEGG ortholog groups [KOs]) of each sample for **(A)** Human immune proteins and microbial KOs, **(B)** microbial KOs, **(C)** human proteins, **(D)** human immune proteins. Boxplots showing the dispersion of these distances, as assessed with the *betadisper* function of the vegan package.

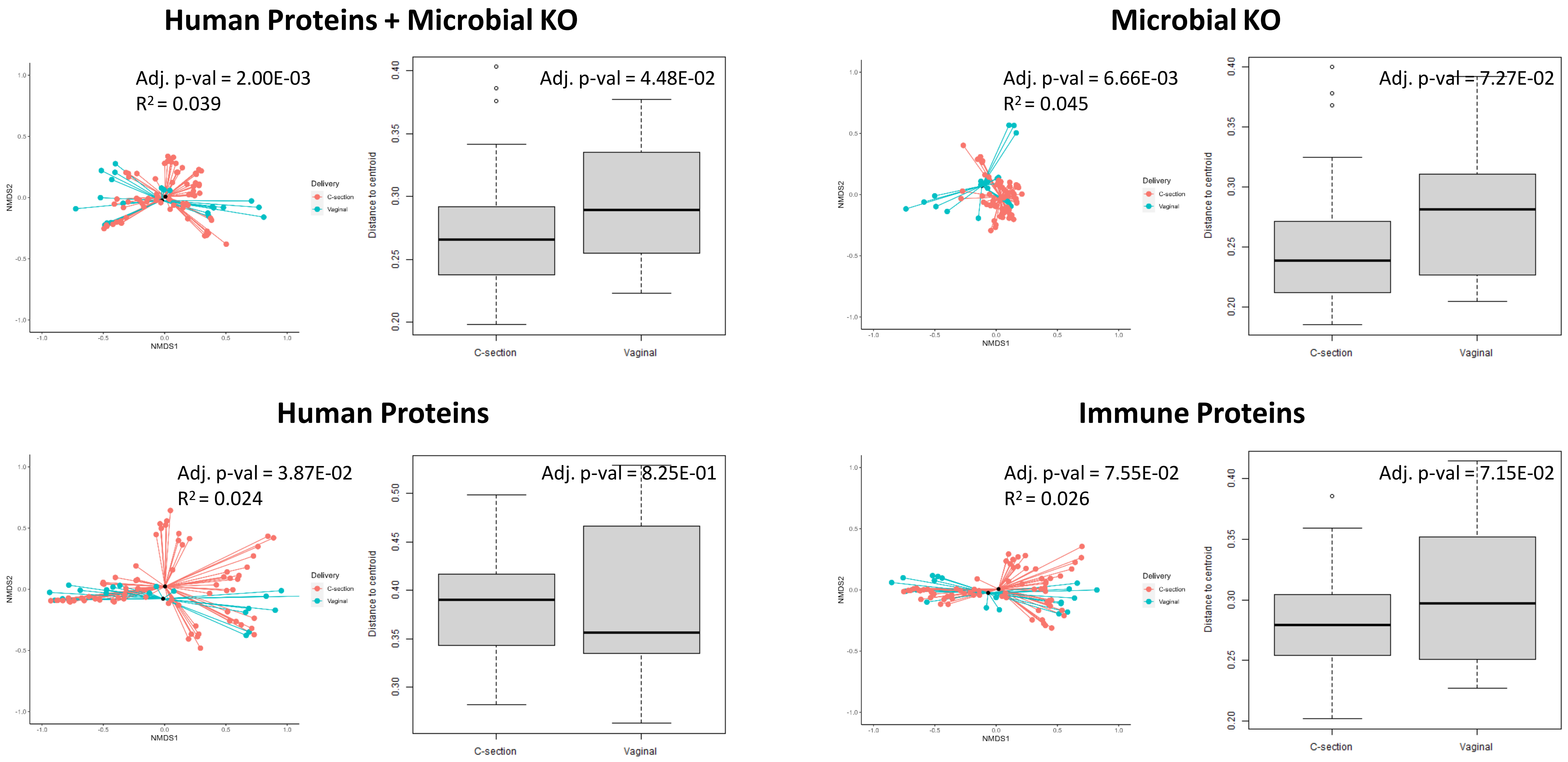

Supplemental Figure 1 5. Functional β-diversity by proteins source for the 91 samples in the study based on the delivery mode of the infant. Non-metric multidimensional scaling (NMDS) of Bray–Curtis distances for the collective functionality (based on presence/absence and abundance of proteins or KEGG ortholog groups [KOs]) of each sample for **(A)** Human immune proteins and microbial KOs, **(B)** microbial KOs, **(C)** human proteins, **(D)** human immune proteins. Boxplots showing the dispersion of these distances, as assessed with the *betadisper* function of the vegan package.

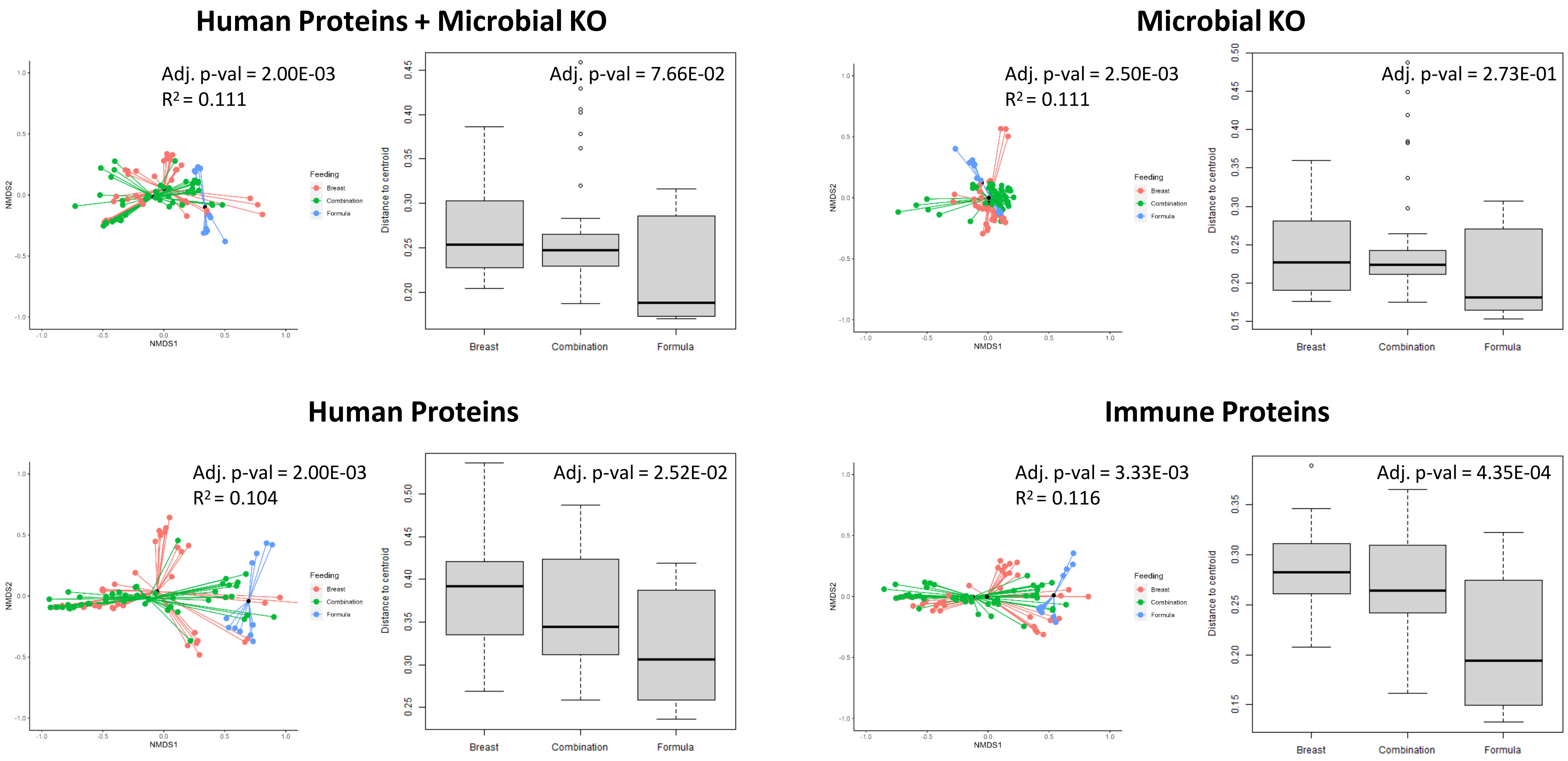

Supplemental Figure 16. Functional β-diversity by proteins source for the 91 samples in the study based on the feeding type of the infant at the time of sample collection. Non-metric multidimensional scaling (NMDS) of Bray–Curtis distances for the collective functionality (based on presence/absence and abundance of proteins or KEGG ortholog groups [KOs]) of each sample for **(A)** Human immune proteins and microbial KOs, **(B)** microbial KOs, **(C)** human proteins, **(D)** human immune proteins. Boxplots showing the dispersion of these distances, as assessed with the *betadisper* function of the vegan package.

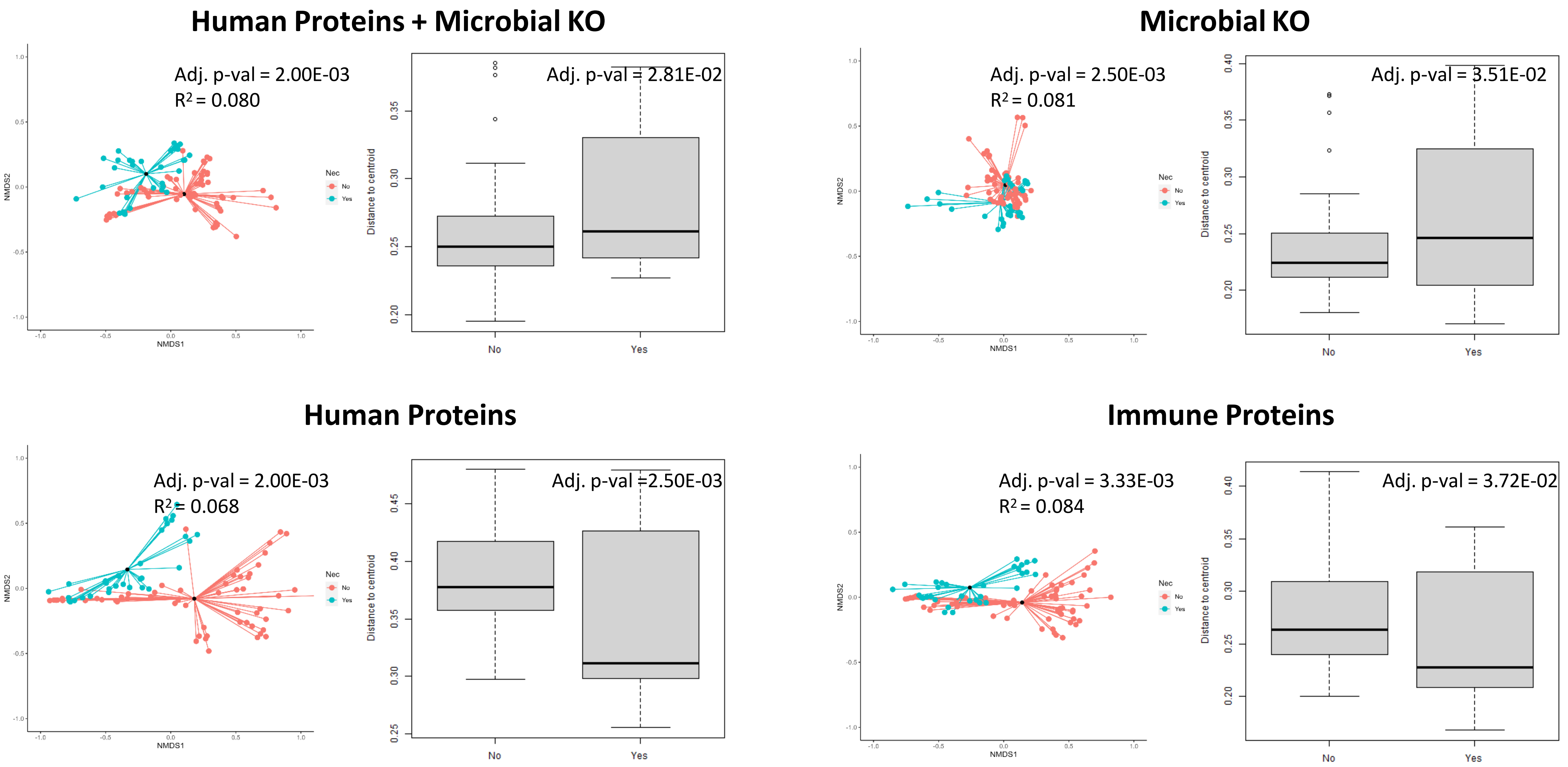

Supplemental Figure 17. Functional β-diversity by proteins source for the 91 samples in the study based on the diagnosis of necrotizing enterocolitis (NEC) for each infant during the course of the study. Non-metric multidimensional scaling (NMDS) of Bray–Curtis distances for the collective functionality (based presence/absence and abundance of proteins or KEGG ortholog groups [KOs]) of each sample for **(A)** Human immune proteins and microbial KOs, **(B)** microbial KOs, **(C)** human proteins, **(D)** human immune proteins. Boxplots showing the dispersion of these distances, as assessed with the *betadisper* function of the vegan package.

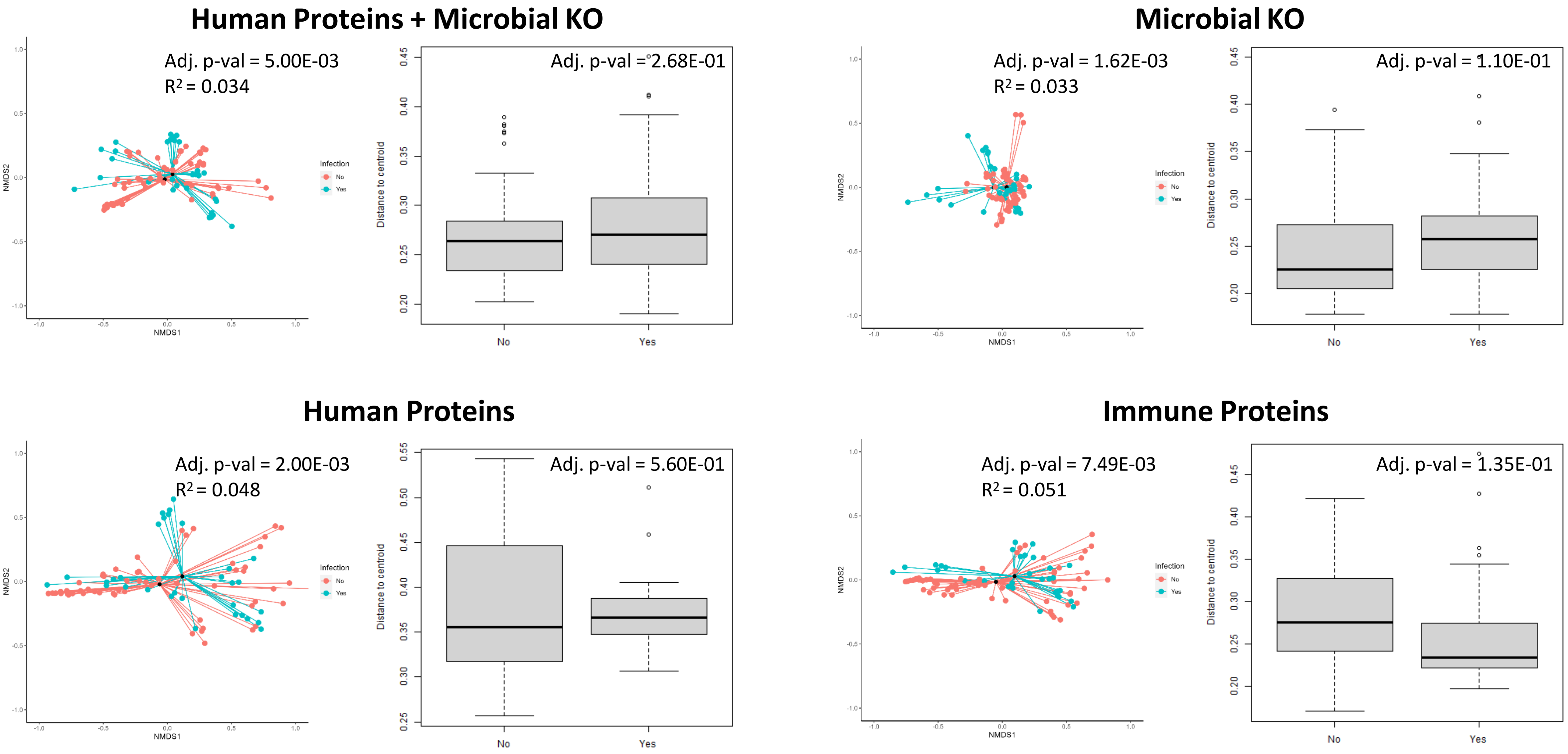

Supplemental Figure 18. Functional β-diversity by proteins source for the 91 samples in the study based on the diagnosis of infection for each infant during the course of the study. Non-metric multidimensional scaling (NMDS) of Bray–Curtis distances for the collective functionality (based on presence/absence and abundance of proteins or KEGG ortholog groups [KOs]) of each sample for **(A)** Human immune proteins and microbial KOs, **(B)** microbial KOs, **(C)** human proteins, **(D)** human immune proteins. Boxplots showing the dispersion of these distances, as assessed with the *betadisper* function of the vegan package.

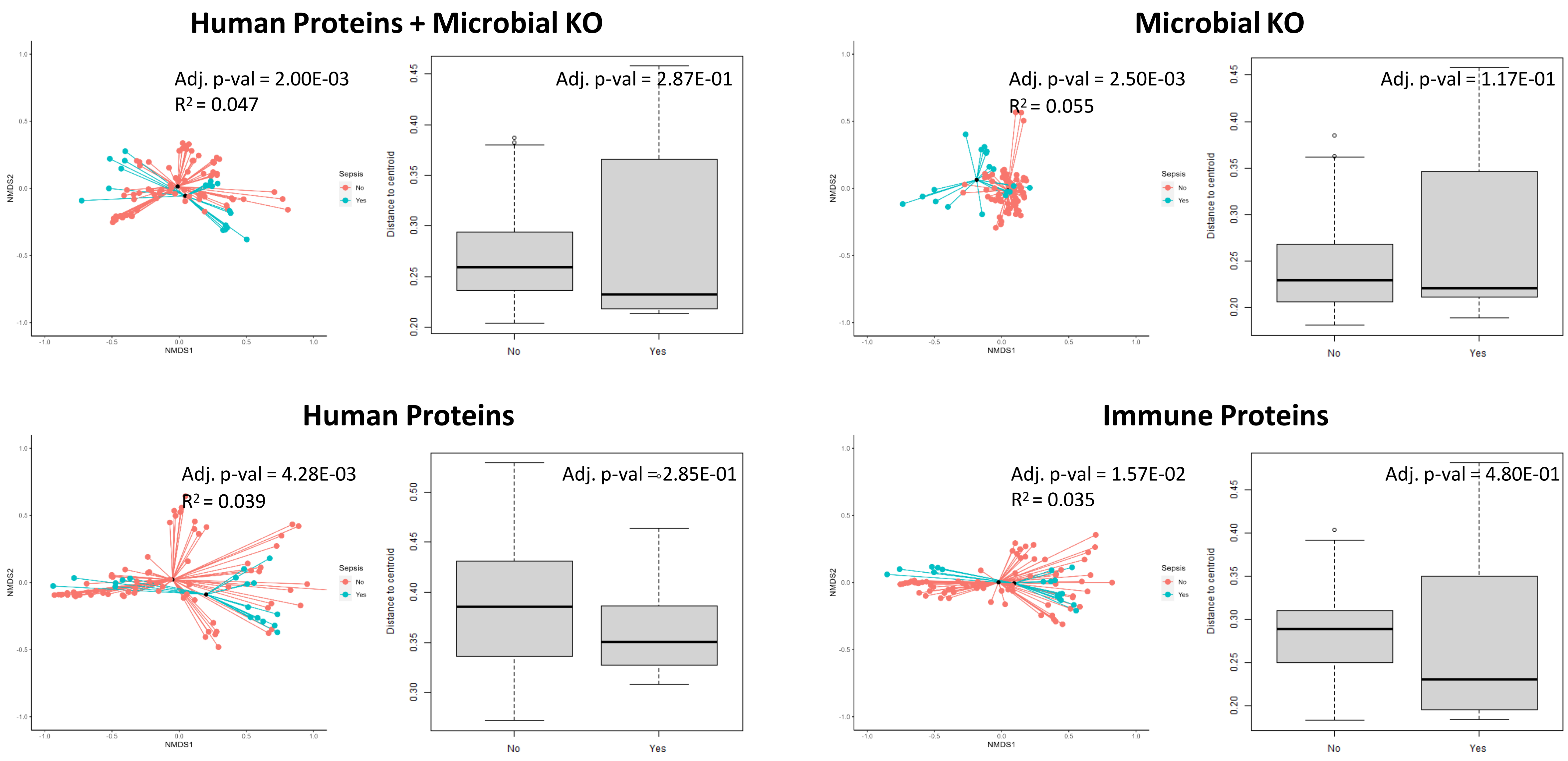

Supplemental Figure 19. Functional β-diversity by proteins source for the 91 samples in the study based on the diagnosis of sepsis for each infant during the course of the study. Non-metric multidimensional scaling (NMDS) of Bray–Curtis distances for the collective functionality (based on presence/absence and abundance of proteins or KEGG ortholog groups [KOs]) of each sample for **(A)** Human immune proteins and microbial KOs, **(B)** microbial KOs, **(C)** human proteins, **(D)** human immune proteins. Boxplots showing the dispersion of these distances, as assessed with the *betadisper* function of the vegan package.

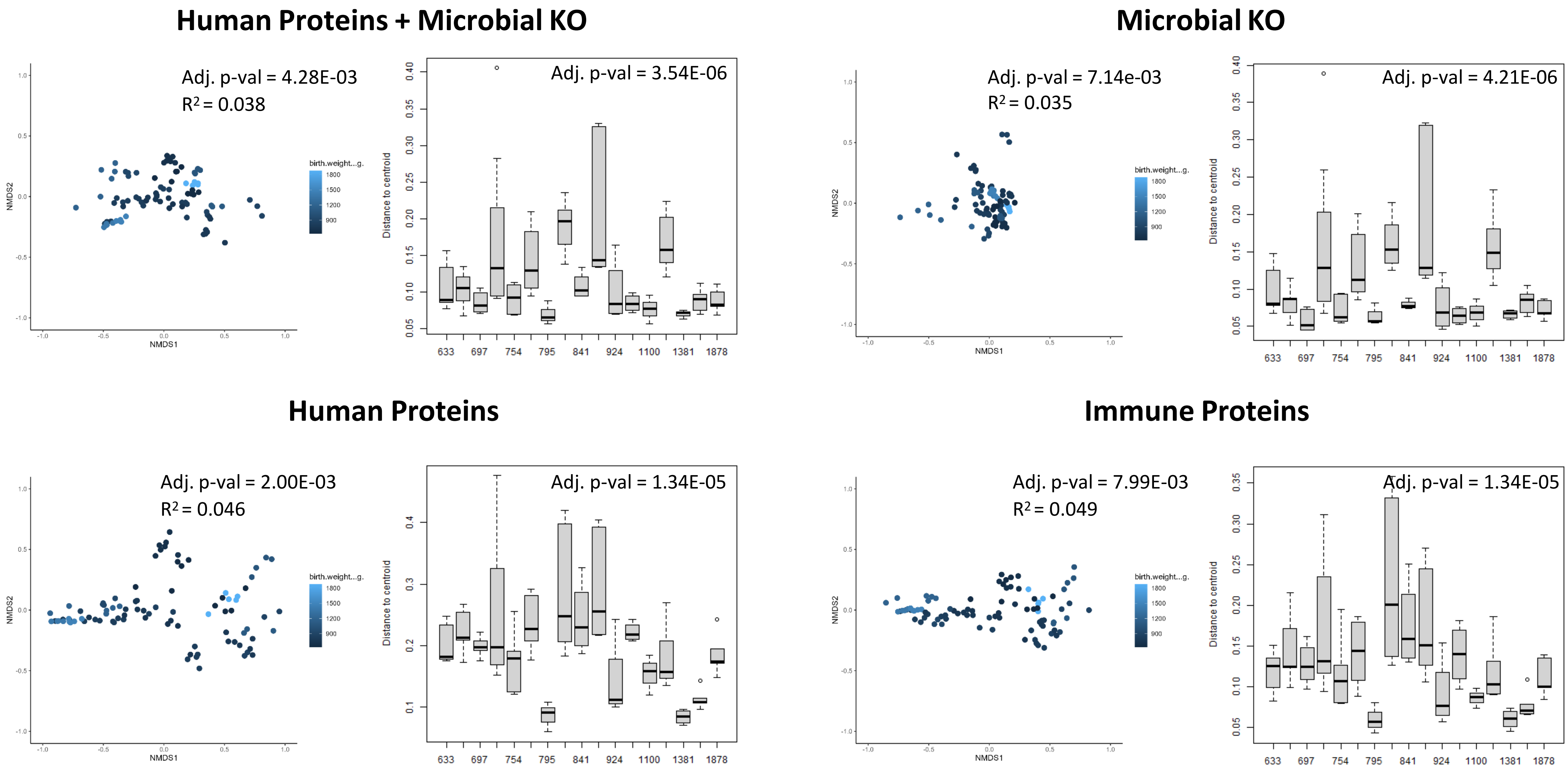

Supplemental Figure 20. Functional β-diversity by proteins source for the 91 samples in the study based on birth weight (g) of each infant. Non-metric multidimensional scaling (NMDS) of Bray–Curtis distances for the collective functionality (based on presence/absence and abundance of proteins or KEGG ortholog groups [KOs]) of each sample for **(A)** Human immune proteins and microbial KOs, **(B)** microbial KOs, **(C)** human proteins, **(D)** human immune proteins. Boxplots showing the dispersion of these distances, as assessed with the *betadisper* function of the vegan package.
